# The *MET13* Methylenetetrahydrofolate Reductase Gene Is Essential for Infection-Related Morphogenesis in the Rice Blast Fungus *Magnaporthe oryzae*

**DOI:** 10.1371/journal.pone.0076914

**Published:** 2013-10-07

**Authors:** Xia Yan, Yawei Que, Hong Wang, Congcong Wang, Ya Li, Xiaofeng Yue, Zhonghua Ma, Nicholas J. Talbot, Zhengyi Wang

**Affiliations:** 1 State Key Laboratory for Rice Biology, Biotechnology Institute, Zhejiang University, Hangzhou, Zhejiang, China; 2 School of Biosciences, University of Exeter, Exeter, Devon, United Kingdom; Rutgers University, United States of America

## Abstract

Methylenetetrahydrofolate reductases (MTHFRs) play a key role in the biosynthesis of methionine in both prokaryotic and eukaryotic organisms. In this study, we report the identification of a novel T-DNA-tagged mutant WH672 in the rice blast fungus *Magnaporthe oryzae*, which was defective in vegetative growth, conidiation and pathogenicity. Analysis of the mutation confirmed a single T-DNA insertion upstream of *MET13*, which encodes a 626-amino-acid protein encoding a MTHFR. Targeted gene deletion of *MET13* resulted in mutants that were non-pathogenic and significantly impaired in aerial growth and melanin pigmentation. All phenotypes associated with *Δmet13* mutants could be overcome by addition of exogenous methionine. The *M. oryzae* genome contains a second predicted MTHFR-encoding gene, *MET12*. The deduced amino acid sequences of Met13 and Met12 share 32% identity. Interestingly, *Δmet12* mutants produced significantly less conidia compared with the isogenic wild-type strain and grew very poorly in the absence of methionine, but were fully pathogenic. Deletion of both genes resulted in *Δmet13Δmet12* mutants that showed similar phenotypes to single *Δmet13* mutants. Taken together, we conclude that the MTHFR gene, *MET13*, is essential for infection-related morphogenesis by the rice blast fungus *M. oryzae*.

## Introduction

Methylenetetrahydrofolate reductase (MTHFR; EC 1.5.1.20) catalyzes the reduction of 5, 10-methylenetetrahydrofolate (CH_2_-THF) to 5-methyltetrahydrofolate (CH_3_-THF), which is required for methionine biosynthesis. In humans, deficiency of MTHFR is the most common genetic defect in folate metabolism, which results in hyperhomocysteinemia, homocystinuria, and hypomethionemia [1]. Elevated levels of homocysteine, due to MTHFR deficiency, are also associated with increased risks of vascular disease and neural tube defects [1,2]. Genes encoding MTHFRs of many species, including bacteria, fungi, plants and mammals, have previously been identified and characterized [1,3-5]. Most fungal species appear to have two MTHFR encoding genes, such as *MET12* and *MET13* in *Saccharomyces cerevisiae* [1], *MET11* and *MET9* in *Schizosaccharomyces pombe* [6], *META* and *METF* in *Aspergillus nidulans* [7], and *FgMET13* and *FgMET12* in *Fusarium graminearum* [8]. In *S. cerevisiae*, disruption of *MET13* causes methionine auxotrophy, while disruption of *MET12* causes no detectable phenotype [1,2]. However, in *A. nidulans*, disruption of either *METF* (*MET13* homologue) or *META* (*MET12* homologue) results in methionine auxotrophy [7]. Targeted gene replacement of either *F. graminearum FgMET13* or *FgMET12* also leads to methionine auxotrophy and affects pigment biosynthesis [8]. However, the role of MTHFRs in plant infection by pathogenic fungi has not been explored in detail.

The ascomycete fungus *Magnaporthe oryzae* is the causal agent of rice blast, the most destructive disease of rice worldwide [9,10]. Rice blast infections are initiated by attachment and germination of conidia on the rice leaf surface. A dome-shaped infection structure, called an appressorium, forms at the tip of a short germ tube and uses turgor to drive a rigid penetration peg through the rice leaf cuticle [10]. After penetration, the fungus forms a primary invasive hypha that further differentiates into bulbous infection hyphae within infected host plant cells. *M. oryzae* grows biotrophically without killing infected plant cells during the early stages of infection. Necrotic disease lesions develop on rice plants after 3-5 days and abundant conidia are produced from lesions during the late stages of infection to re-initiate successive rounds of plant infection. Due to its socioeconomic impact and genetic tractability, *M. oryzae* has been utilized as a model fungal pathogen for understanding the molecular basis of plant-fungus interactions [11-16]. In the past decade, there have been extensive studies on molecular mechanisms that regulate plant infection-related morphogenesis and pathogenicity in *M. oryzae*. Many genes involved in signaling or metabolic pathways, required for fungal growth, conidiation, appressorium formation, penetration and pathogenicity, have been identified and characterized [14,16-27]. However, there are still fundamental gaps in our knowledge regarding the contribution of fundamental pathways, such as amino acid biosynthesis, to the establishment of rice blast disease.

Recently, we carried out a large-scale *Agrobacterium tumefaciens*-mediated transformation (ATMT) screen of *M. oryzae* to identify insertional mutants defining novel genes required for pathogenicity. Several important genes, required for regulating different stages of infection-related morphogenesis in *M. oryzae*, have been identified from this initial screen [28-30]. Here, we present the identification and characterization of *M. oryzae*, *MET13*, which putatively encodes a MTHFR required for pathogenicity. Targeted gene deletion mutants of *MET13* were methionine auxotrophs, consistent with the predicted role for *MET13* in methionine biosynthesis. Additionally, aerial growth and colony pigmentation were affected by absence of the gene. *MET13* is therefore essential for infection-related morphogenesis and pathogenicity in the rice blast fungus.

## Results

### Identification of a non-pathogenic mutant WH672

**Figure 1:**
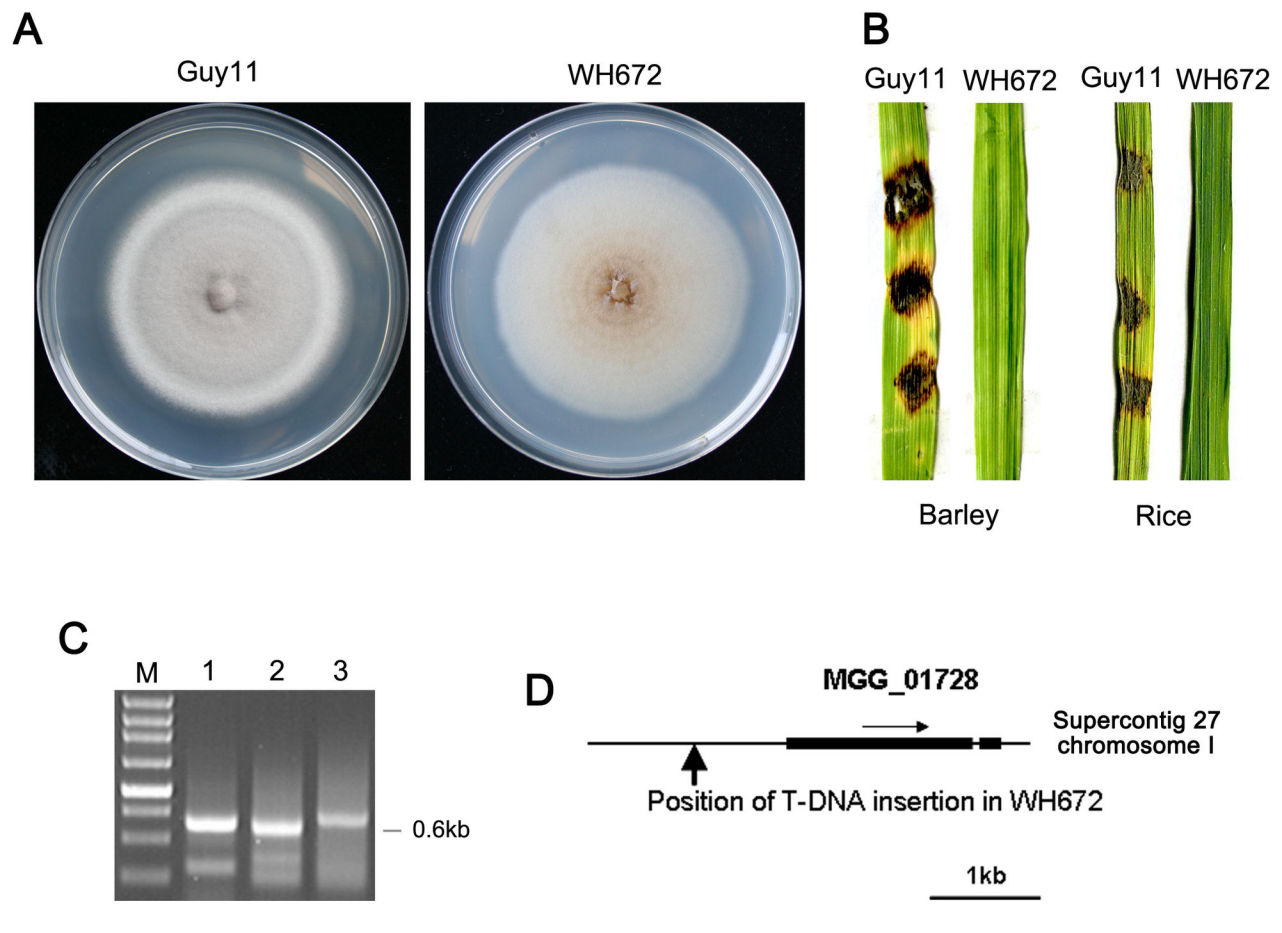
Identification of the non-pathogenic mutant, WH672, and the T-DNA-tagged gene. (**A**) Colonies of the wild type strain Guy11 and T-DNA insertional mutant WH672 on CM medium. (**B**) Barley and rice leaf segments were inoculated with the mycelium plugs from Guy11 and WH672 cultures. (**C**) Three round amplifications of 0.6 kb genomic DNA flanking right site of the integration T-DNA in WH672 using a hiTAIL PCR approach. (**D**) Position of T-DNA insertion in the WH672 mutant strain. Arrow indicates the T-DNA insertion positions in *MGG_01728*. Thick boxes represent the coding regions and the thin line joining these coding regions indicates position of the intron.

We carried out ATMT of the wild-type *M. oryzae* strain Guy11 [31] using a binary vector to obtain insertional mutants displaying defects in pathogenesis. During this screen, we identified a mutant, WH672, which grew poorly on complete medium (hereafter called CM) [32], and did not produce melanin pigmented hyphae (Figure 1A; Table S1). Interestingly, the mutant was unable to sporulate and was also incapable of causing rice blast disease following inoculation of susceptible barley or rice leaves with hyphae (Figure 1 B). DNA gel blot analysis showed that a single T-DNA integration event had occurred in the genome of WH672 (not shown). We therefore decided to perform further characterization of the mutant to determine its role in pathogenicity.

### Identification of the T-DNA tagged gene *MET13* in WH672

To identify the T-DNA integration site in the WH672 mutant, a 0.6 kb genomic DNA flanking region of the integrated T-DNA (Figure 1C) was obtained using a hi-TAIL PCR strategy [33], and then sequenced. Bioinformatics analysis revealed that the T-DNA insertion site was situated at 815 bp upstream of the translational start site of a gene, MGG_01728.6 (XP_363802), located on supercontig 27 of chromosome I (Figure 1D), which putatively encodes a Methylenetetrahydrofolate reductase (MTHFR 1). We named the T-DNA tagged gene *MET13*, because it encodes a protein showing 46.59% identity to *S. cerevisae* Met13. To confirm the position and size of the introns of *M. oryzae MET13*, reverse transcription-PCR (RT-PCR) was carried out using the primers CDS13F and CDS13R (Table S2) and the cDNA sequence and genome sequence were compared. This confirmed that *MET13* has an open reading frame of 1,878 bp interrupted by a single intron (67 bp) and putatively encodes a 626 aa protein, which is 100% identical to the protein sequence predicted by automated annotation of the *M. oryzae* genome sequence [34] (Figure 1D). A local BLASTP search with *M. oryzae* Met13 revealed that the fungus has another gene *MET12* (MGG_08171.6), that putatively encodes a second MTHFR (MTHFR2) with 690 aa, which is homologous to *S. cerevisae* Met12 (42.84% amino acid identity). *MET12* has an open reading frame of 2,070 bp without introns, confirmed by RT-PCR using the primers CDS12F and CDS12R (Table S2). Alignment of *M. oryzae* Met13 and Met12 is shown in Figure S1 and they share 31.98% identity at the amino acid level. Additionally, Met13 and Met12 exhibit high levels of amino acid identity to corresponding MTHFRs from related fungi, such as *Neurospora crassa* (75.28% and 71.61%), *Gibberella zeae* (73.69% and 69.55%) and *A. nidulans* (62.18% and 56.41%). Phylogenetic analysis of the putative homologues of Met13 and Met12 from several fungal species is shown in Figure S2. These MTHFRs could be divided into two groups: MTHFR1 and MTHFR2, indicating the two MTHFRs may have originated independently.

### *MET13* is crucial for mycelium pigment and virulence

**Figure 2:**
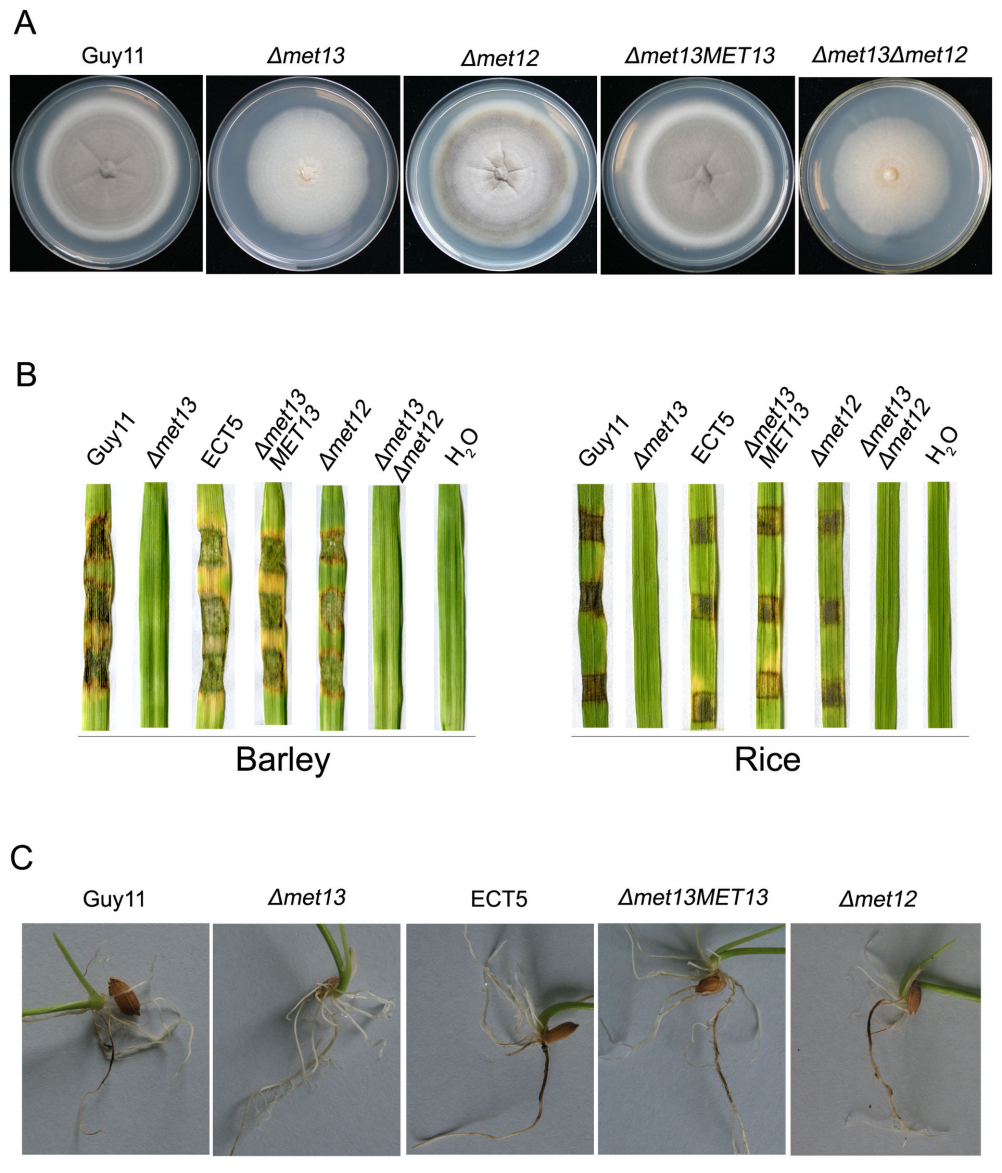
Colony growth and pathogenicity assays of *Δmet12*, *Δmet13* and *Δmet13Δmet12* mutants. (**A**) Colonies of different strains. (**B**) Barley and rice segments inoculated with mycelium plugs from the wild-type strain Guy11, *Δmet13* (K56), ectopic transformant (ECT5), *Δmet13Met13* (C3), *Δmet12* (K12-7) and *Δmet13Δmet12* (DK7). H_2_O was used as the negative control. (**C**) Rice root infection assays. Photographs were taken 5 days after inoculation.

To verify the phenotypes associated with WH672 were caused by the insertion mutation, we performed targeted gene deletion of *MET13* using the gene replacement vector pMET13-KO (Figure S3A). Four targeted gene replacement mutants, K56, K73, K85 and K96 (Table S1), were identified by PCR analysis from 96 transformants using primers CDS13F and CDS13R (Table S2; Figure S3A). Gene replacement of the K56 and K73 mutants was further confirmed by Southern blot analysis and PCR amplification using primers F13 and R13 (Table S2; Figure S3B and C). Using a similar strategy, two targeted gene deletion mutants of *MET12*, K12-7 and K12-13, were obtained (Figure S3 D and E). The four *Δmet13* or two *Δmet12* mutants showed consistent phenotypes, although only K56 (*Δmet13*) and K12-7 (*Δmet12*) are described below. Consistent with mutant WH672, the *Δmet13* mutant formed colonies that were poorly pigmented when grown on CM medium and produced very few aerial hyphae when compared with Guy11 (Figure 2A). However, the *Δmet12* mutant grew normally and formed colonies that were more similar in appearance to Guy11, albeit slightly less pigmented (Figure 2A). In order to further investigate the roles of *MET13* and *MET12* in development and virulence of *M. oryzae*, three *Δmet13Δmet12* double mutants (DK7, DK13 and DK18) were generated (Table S1; Figure S3F). The *Δmet13Δmet12* mutants (DK7) formed similar colonies to single *Δmet13* mutants on CM medium (Figure 2A), indicating that *MET13* may play more important roles in aerial growth and melanization than *MET12* in *M. oryzae*.

To test for the ability of the mutants to cause rice blast disease, a modified assay was used because *Δmet13* and *Δmet13Δmet12* mutants were unable to sporulate. Mycelial plugs were removed from the edges of 10 day-old colonies and used to inoculate plants. The *Δmet13*, *Δmet12* and *Δmet13Δmet12* mutants were used to inoculate leaves of the susceptible barley cultivar Golden Promise and rice cultivar CO-39 using a cut-leaf assay as shown in Figure 2B. The *Δmet13* mutant was completely non-pathogenic on both susceptible barley and rice leaves (Figure 2B), even when leaf surfaces were abraded, consistent with the non-pathogenic phenotype of WH672 and confirming that *MET13* is necessary for virulence of *M. oryzae*. Similarly, the *Δmet13Δmet12* mutants were also non-pathogenic (Figure 2B). However, the *Δmet12* mutant was full pathogenic on both barley and rice (Figure 2B). Similar results were observed when mycelium was harvested from liquid CM cultures and used to inoculate barley or rice leaves (not shown). Root infection assays showed that the *Δmet13* mutants, but not *Δmet12* mutants, were also defective in root infection on rice seedlings (Figure 2C). We conclude that *M. oryzae MET13* is essential for virulence in *M. oryzae*.

### *MET13* is essential for conidiation and appressorium formation by *M. oryzae*

**Figure 3:**
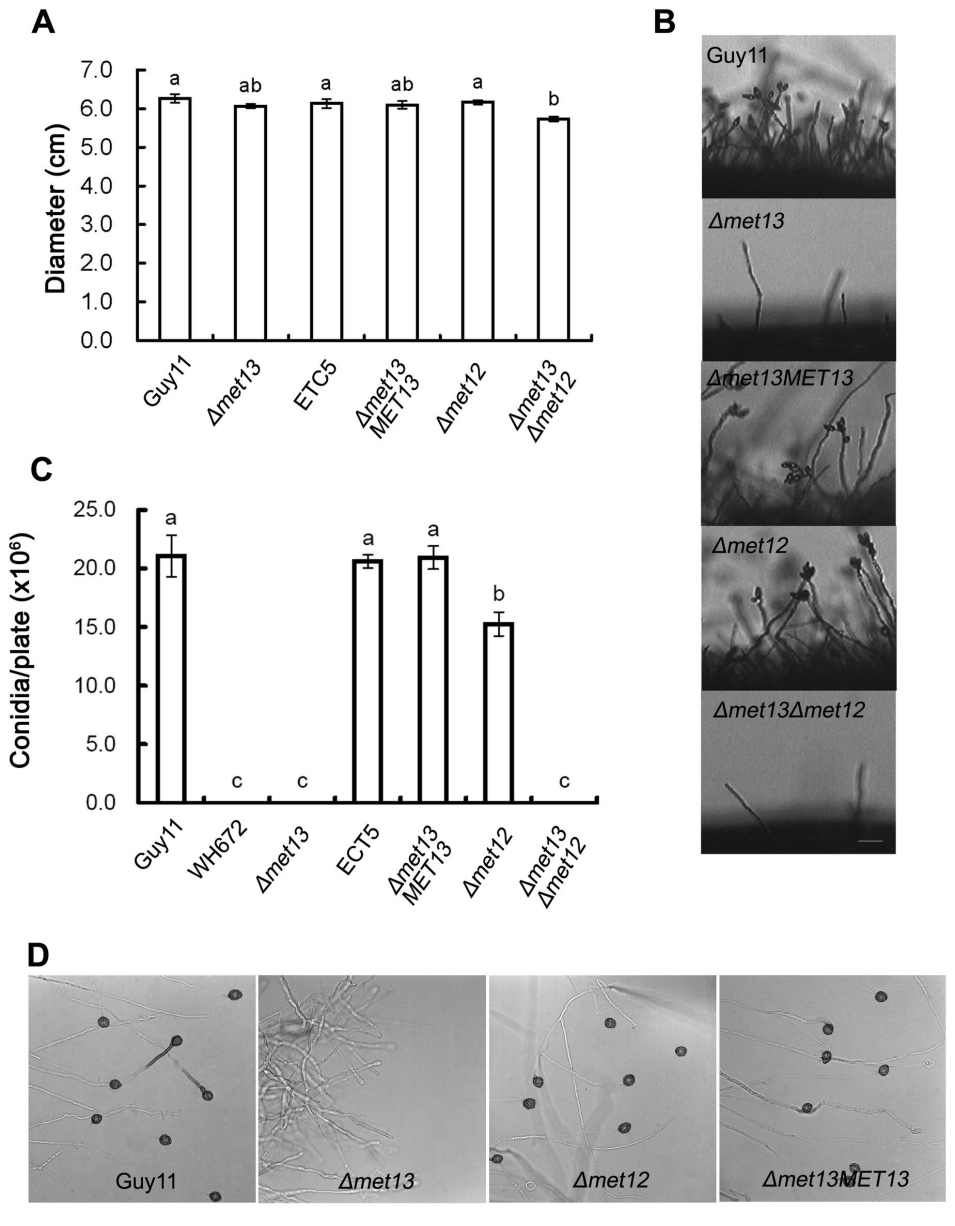
Phenotypic analysis of *Δmet13* and *Δmet12* mutants. (**A**) Bar chart showing colony diameters of the wild-type strain Guy11, *Δmet13* (K56), ectopic transformant (ECT5), *Δmet13MET13* (C3), *Δmet12* (K12-7) and *Δmet13Δmet12* (DK7) on CM medium at 25°C for 10 days. Error bars represent the standard deviation. Lower case letters indicate significant differences at *P* = 0.05. (**B**) Microscopic observation of conidial development. Aerial hyphae were significantly reduced and conidia were not observed in the *Δmet13* and *Δmet13Δmet12* mutants. The wild-type strain Guy11 and the complementation strain (Δmet13MET13) formed normal conidiophores and numerous conidia. All the tested strains grown on CM medium for 4 days were examined by light microscopy. Bar = 20 μm. (**C**) Bar chart showing the conidial production. The WH672, *Δmet13* and *Δmet13Δmet12* mutants were unable to produce any conidia, while the *Δmet12* muatnt produced significantly less conidia than Guy11, ECT5 and *Δmet13MET13* on CM medium at 25°C for 12 days. Error bars represent standard deviation. (**D**) Appressoria were formed by the hyphal tips of Guy11, *Δmet13*, *Δmet12* and *Δmet13MET13* on hydrophobic surfaces. After 24 h incubation at 25°C in the dark, numerous appressoria were produced by Guy11, *Δmet12* and *Δmet13MET13*, however, no appressoria were observed after inoculation of *Δmet13*. Bar = 20μm.

Phenotypic analysis showed that the *Δmet13* and *Δmet12* mutants were not significantly reduced in radial growth, forming colonies with diameters of 6.07 ± 0.06 cm and 6.17 ± 0.06 cm after 10-days incubation on CM at 25°C, respectively. However, the *Δmet13Δmet12* mutants formed smaller colonies with a diameter of 5.73 ± 0.06 cm (*P*<0.05), compared with 6.27 ± 0.12 cm in the wild-type strain Guy11 (Figure 2A and Figure 3A). The ability to form asexual spores was evaluated by carefully washing the surface of 12-day-old cultures. WH672, *Δmet13* and *Δmet13Δmet12* mutants were unable to produce conidia and formed very few aerial hyphae, whereas Guy11 produced 21.08 ± 1.79×10^6^ spores per plate (Figure 3B and C). The *Δmet12* mutant was able to sporulate but produced significantly fewer conidia 15.27 ± 1.00 × 10^6^ per plate. *MET13* is therefore important for aerial hypha formation and conidiation in *M. oryzae*.

To determine whether deletion of *MET13* had an effect on appressorium formation, we harvested mycelium of the *Δmet13*, *Δmet12* and *Δmet13Δmet12* mutants from liquid CM culture. Hyphae were incubated on hydrophobic surfaces and appressoria allowed to form. Guy11 and the *Δmet12* mutant formed appressoria normally, whereas the *Δmet13* mutant was unable to form appressoria (Figure 3D). Similarly, the WH672 and *Δmet13Δmet12* mutants were unable to form appressoria from mycelium under the same conditions (not shown). The *Δmet12* mutant was also able to form appressoria when conidia were germinated on hydrophobic surfaces (data not shown). These results suggest that *MET13* is required for appressorium formation by *M. oryzae*.

### Deletion of *MET13* results in the inability of *M. oryzae* to grow on minimal growth medium

**Figure 4:**
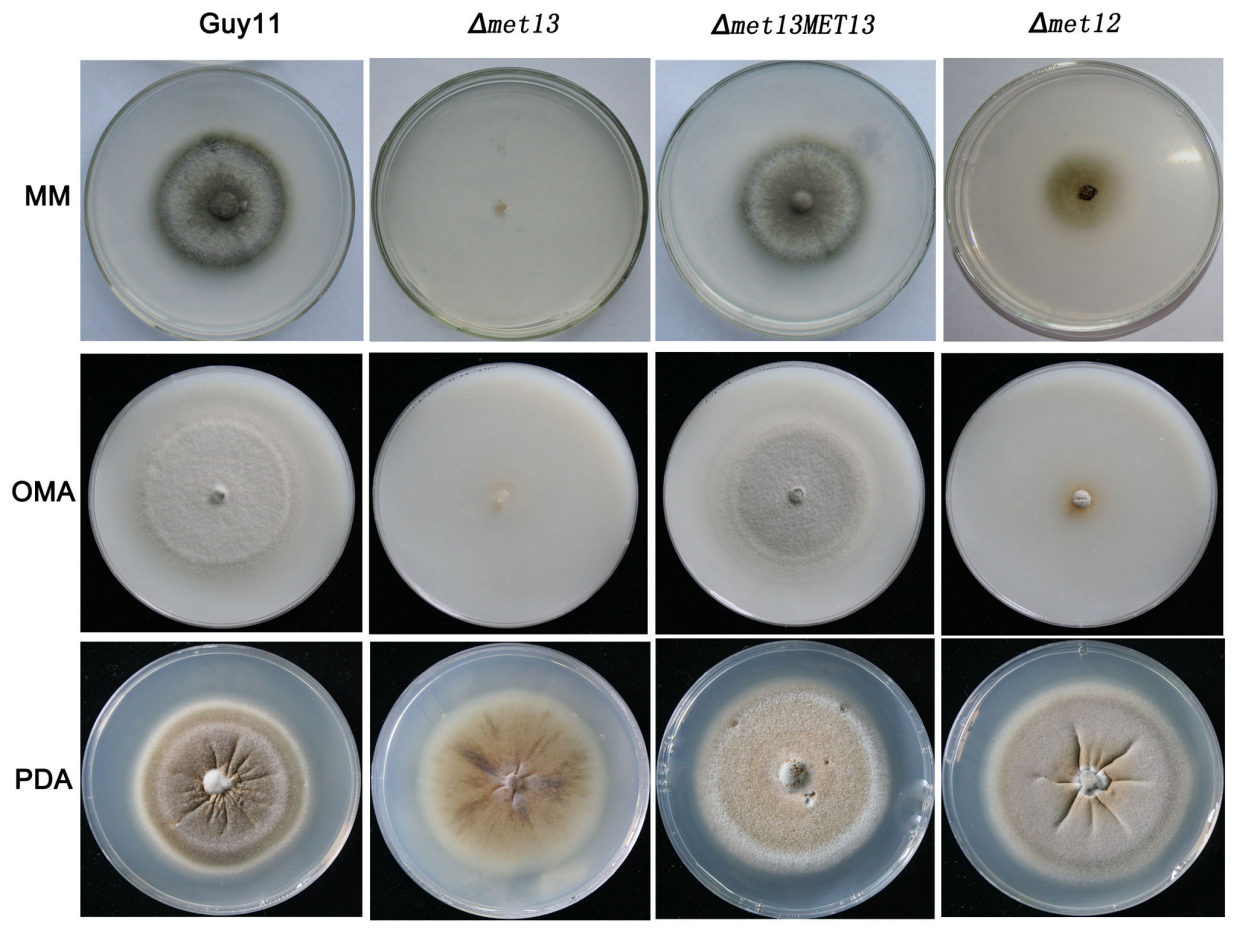
Growth of *Δmet13*, *Δmet13MET13* and *Δmet12* on different medium. The wild-type strain Guy11, *Δmet13* (K56), *Δmet13MET13* (C3) and *Δmet12* (K12-7) were grown on MM, OMA and PDA media at 25°C for 10 days. The *Δmet13* mutant was unable to grow and the growth of the *Δmet12* mutant was significantly restricted on MM and OMA medium.

Deletion of *MET13* resulted in highly reduced aerial hyphae development and pigment production following growth on CM medium (Figure 2A). To investigate the growth of *Δmet13* and *Δmet12* mutants, each strain was grown on alternative growth medium, including minimal medium (MM), oatmeal agar (OMA) and potato dextrose agar (PDA). Interestingly, deletion of *MET13* completely prevented vegetative growth on MM and OMA media, and the growth of the *Δmet12* mutant was also significantly restricted, although both mutants could grow on PDA medium (Figure 4). The growth defects of the mutants on MM and OMA are likely due to the restricted amino acid content of these growth media.

To determine the role of *MET13* and *MET12* in sexual reproduction, the wild type strain Guy11 (*MAT1-2*) and the *Δmet13*, *Δmet12* and *Δmet13Δmet12* double mutants were crossed with a standard tester strain TH3 (*MAT1-1*) [35] of *M. oryzae* to allow perithecium production. After four weeks, junctions between mated individuals were examined for the presence of perithecia. Numerous perithecia were observed at the junctions of crosses between Guy11 and TH3, but no perithecia were formed by the crosses of *Δmet13* × TH3 or *Δmet13Δmet12* × TH3, even when the incubation time was extended (Figure S4). Additionally, only a few perithecia were observed from the cross between *Δmet12* and TH3 (Figure S4). These results suggest that both *MET13* and *MET12* are required for fertility and development of fruiting bodies and sexual spores by *M. oryzae*.

### Re-introduction of *MET13* into a *Δmet13* mutant restores all phenotypes

To ensure that the phenotypic differences observed in *Δmet13* mutants were associated with the gene replacement event, we carried out phenotypic analysis of the complementation transformants C3, C5 and C6 in which the full length *MET13* gene was re-introduced (Table S1). All three transformants exhibited full virulence to barley and rice by spray-inoculation or using cut-leaf assays. All phenotypes of *Δmet13* mutants, including vegetative growth, sporulation and appressorium formation, were fully complemented by re-introduction of the gene (Figures 2-4; Figure S4). However, GFP fluorescence was not observed in hyphae, conidia and appressoria of the complementation transformants, implicating low Met13-GFP expression during vegetative growth and morphological differentiation of *M. oryzae*. Additionally, phenotypes associated with *Δmet12* mutants were also fully restored by re-introducing the full length *MET12* gene into the mutants (not shown).

### *Δmet13* mutant phenotypes can be overcome by addition of exogenous methionine

**Figure 5:**
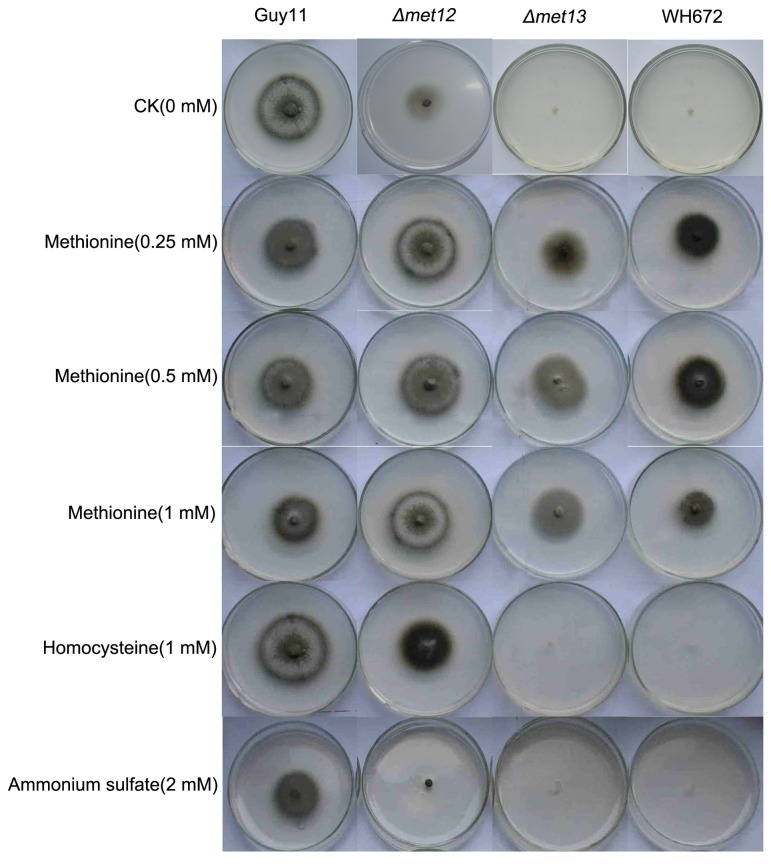
Growth of the wild-type strain and the mutants on MM with methionine, homocysteine or ammonium sulfate. The wild-type strain Guy11, WH672, *Δmet13* (K56) and *Δmet12* (K12-7) were grown on MM medium at 25°C for 10 days. Concentrations of methionine, homocysteine or ammonium sulfate are as indicated.

To determine whether *Δmet13* mutant phenotypes, such as vegetative growth, mycelium pigment and conidiation, could be rescued by adding exogenous methionine, the WH672, *Δmet13* and *Δmet12* mutants were grown on MM medium with 0, 0.25, 0.5 and 1.0 mM methionine at 25°C for 10 days, respectively. We observed that growth and colony pigmentation of WH672, *Δmet13* and *Δmet12* mutants were restored by methionine (Figure 5). Defects in vegetative growth and mycelium pigment for the *Δmet12* but not *Δmet13* mutants were also partially complemented by adding exogenous homocysteine (Figure 5). However, all mutants were unable to grow on MM medium following supplementation with 2 mM ammonium sulfate (Figure 5). Additionally, we observed that *Δmet13* mutants were able to produce conidia on MM medium upon supplementation with methionine, although the number of conidia produced by the mutants was still significantly less than the wild-type strain (data not shown). These results indicate that both *Δmet12* and *Δmet13* mutants are methionine auxotrophs.

To understand whether conidiation of the *Δmet13* mutants could be restored by exogenous methionine, we added 0.5, 1, 2, 4, 6, 8, or 10 mM methionine to CM medium. Our results showed that the defects in aerial growth and mycelium pigment of the *Δmet13* mutant could all be overcome by adding methionine (Figure S5A). However, melanization of the *Δmet13* mutant was impaired when supplemented with >6 mM methionine (Figure S5A). Supplementation with methionine also partially restored conidiation (Figure S5B). The growth defect of the *Δmet13* mutant in liquid CM medium was also complemented by adding 1 mM methionine (Figure S6). Taken together, the developmental phenotypes associated with *Δmet13* mutants, including vegetative growth, mycelium pigment and conidiation could all be overcome by addition of exogenous methionine.

**Figure 6:**
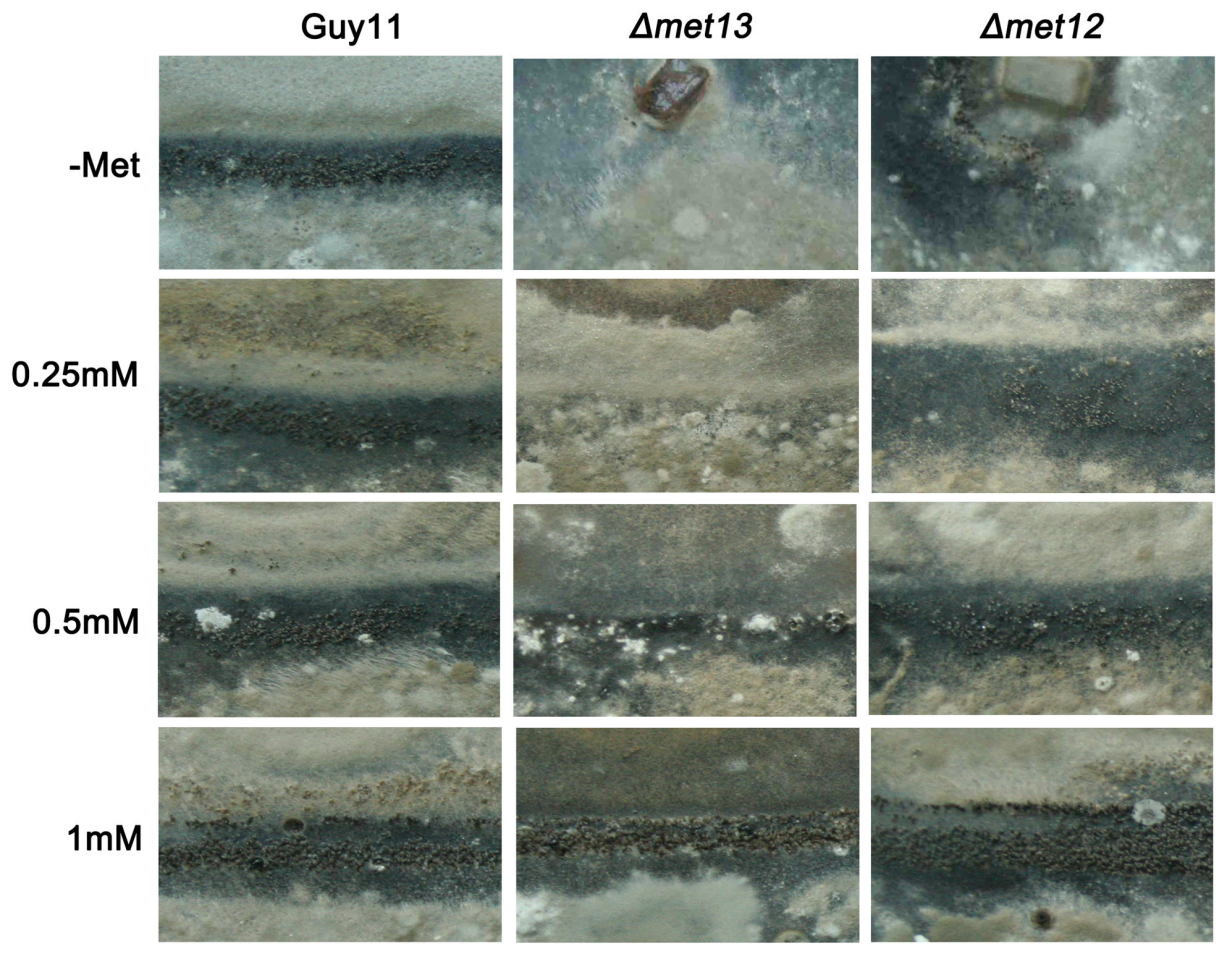
Perithecium production of the *Δmet13* and *Δmet12* mutants on OMA medium with methionine. Concentrations of methionine in OMA medium were indicated. Perithecium production of the *Δmet13* (K56) and *Δmet12* (K12-7) mutants was fully complemented by the supplement of 1 mM methionine.

**Figure 7:**
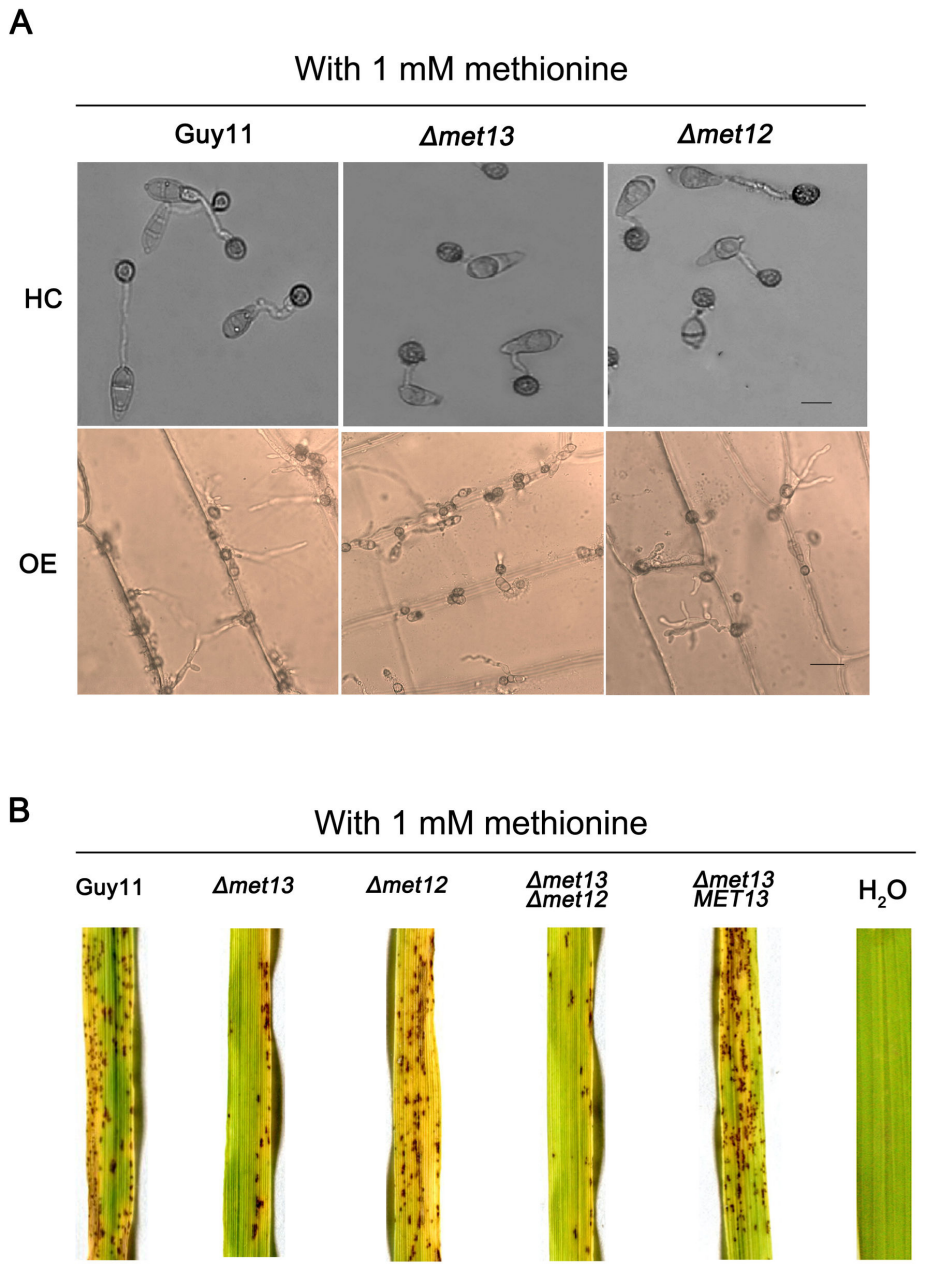
Complementation of appressorium formation, penetration and pathogenicity of the *Δmet13* mutant by adding exogenous methionine. (**A**) Appressoria of the wild-type strain Guy11, K56 (Δmet13) and K12-7(Δmet12) were induced on hydrophobic coverslips (HC) and onion epidermis (OE) surfaces at 25°C in darkness for 24 h. Bar represents 10 μm for top panels and 20 μm for bottom panels. (**B**) Rice infection assays. Conidial suspension of 1 × 10^5^ conidia ml^-1^ were spray-inoculated onto rice seedlings. H_2_O was used as the negative control. Photographs were taken at 5 days after inoculation. For both A and B, conidia of Guy11, K56, C3 (Δmet13MET13), K12-7 and DK7 (Δmet13Δmet12) were harvested from the CM cultures with 2 mM methionine and then diluted in 1 mM methionine solution.

We further tested sexual reproduction of the *Δmet13* mutants on OMA medium following supplementation with methionine. We observed numerous perthecia at the junctions of crosses Guy11 × TH3 and *Δmet12* × TH3 following supplementation with 0.25 mM or 0.5 mM methionine, whereas few perithecia of the cross of *Δmet13* × TH3 was formed (Figure 6). However, numerous perthecia were produced from all crosses when the concentration of methionine increased to 1 mM (Figure 6). Sexual morphogenesis in *Δmet13* mutants can therefore be overcome by exogenous methionine. Similarly, appressorium development could also be restored following methionine supplementation (Figure 7A). However, the penetration rate of the *Δmet13* mutant was only 40.67 ± 7.02%, which was significantly lower than 81.33 ± 4.73% of Guy 11 and 78.67 ± 6.11% of the *Δmet12* mutant. Moreover, invasive haphae formed by the *Δmet13* mutant in onion epidermic cells were shorter than those of the wild type strain (Figure 7A).

To test whether pathogenicity could be restored in the presence of methionine, spores of the *Δmet13* mutant were harvested from the CM cultures containing 2 mM methionine and spores diluted to 1 × 10^5^ spores ml^-1^ in 1 mM methionine solution. Conidial suspensions were then spray-inoculated onto susceptible rice leaves. We observed that the *Δmet13* mutant was able to infect rice leaves and form typical blast lesions, although the virulence of the mutant was moderate compared with Guy11 and *Δmet12* (Figure 7 B). Virulence of the *Δmet13* mutants can therefore be restored by exogenous methionine.

### Deletion of *MET13* results in up-regulation of *MET12* and vice versa

**Figure 8:**
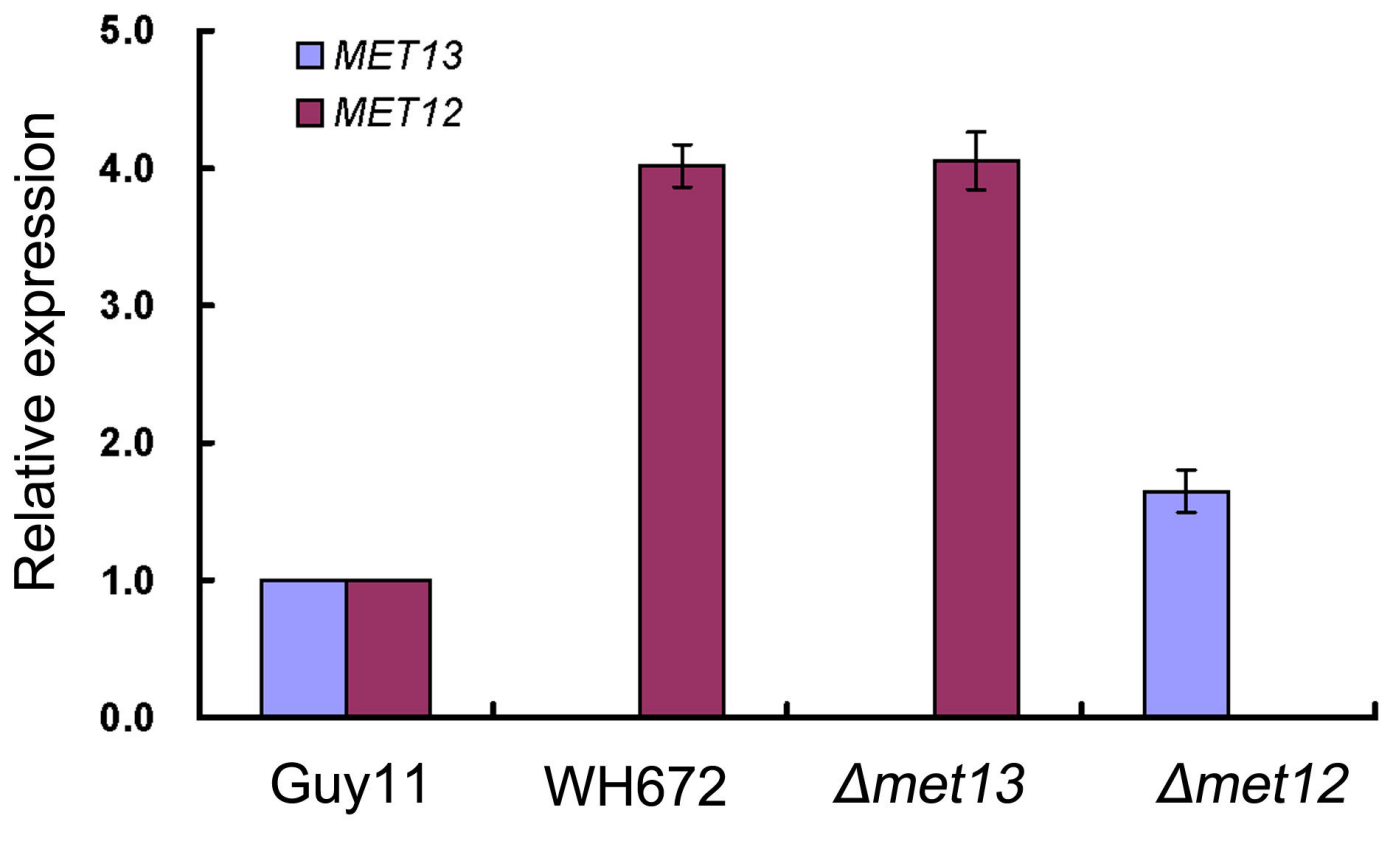
Relative expression of *MET13* and *MET12* in *Δmet13* and *Δmet12* mutants. Measurements of gene transcripts by quantitative RT-PCR analysis were normalized to β-tubulin (MGG_00604.6) and expressed as relative values, with 1 corresponding to Guy11. Error bars represent the standard deviation. Expression of *MET12* in WH672 and K56 (Δmet13) and *MET13* in K12-7*2* (Δmet12) was significantly elevated.

As both *MET12* and *MET13* putatively encode MTHFR enzymes, we decided to investigate whether the expression of each gene was altered upon deletion of the other. We therefore carried out quantitative RT-PCR (qRT-PCR) analysis. As expected, gene expression of *MET13* was not detected in WH672 and *Δmet13* and *MET12* was not expressed in the *Δmet12* mutant (Figure 8). However, expression of *MET13* was 1.6 fold up-regulated in the *Δmet12* mutant and expression of *MET12* was 4.0 fold up-regulated in both WH672 and *Δmet13* mutants (Figure 8). These results are consistent with both genes encoding MTHFR and an attempt to restore methionine biosynthesis by up-regulation of the alternative gene.

### Expression analysis of genes involved in methionine biosynthesis upon deletion of *MET13*

**Figure 9:**
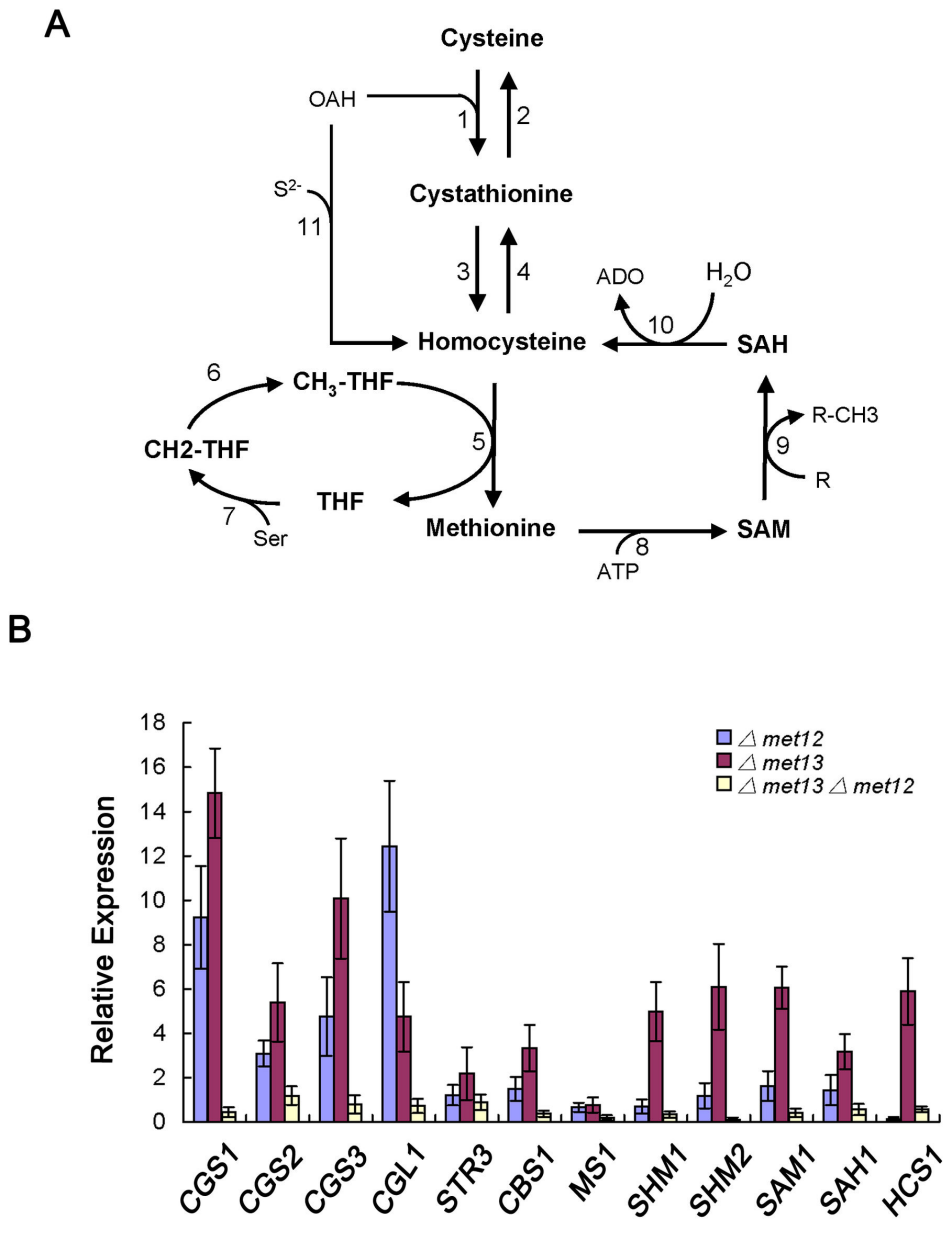
The predicted pathway for methionine biosynthesis in *M. oryzae* and expression of several genes involved in the pathway in *Δmet13*, *Δmet12* and *Δmet13Δmet12* mutants. (**A**) An outline of methionine metabolism in *M*. *oryzae*. The predicted genes and corresponding enzymes: 1. *CGS1* (MGG_03583), *CGS2* (MGG_09292) and *CGS3* (MGG_04764) - cystathionine γ-synthase; 2. *CGL1* (MGG_10380) - cystathionine γ-lyase; 3. *CBL1*/*STR3* (MGG_07074) - cystathionine β-lyase; 4. *CBS1* (MGG_07384) - cystathionine β-synthase; 5. *MS1* (MGG_06712) - methionine synthase; 6. *MET13* (MGG_01728) and *MET12* (MGG_08171) - methylenetetrahydrofolate reductase (MTHFR); 7. *SHM1* (MGG_13781) and *SHM2* (MGG_00923) - serine hydroxymethyltransferase; 8. *SAM1* (MGG_00383) - S-adenosyl-methionine synthase; 9. various methyltransferases; 10. *SAH1* (MGG_05155) - S-adenosyl homocysteine hydrolase; 11. *HCS1* (MGG_07195) - homocysteine synthase. SAM: S-adenosyl methionine; SAH: S-adenosyl homocysteine; OAH: O-acetylhomoserine; ADO: Adenosine; Ser: Serine; R: any methylacceptor substrate. (**B**) Expression of the predicted genes coding enzymes involved in methionine metabolism in the *Δmet13*, *Δmet12* and *Δmet13Δmet12* mutants measured by quantitative reverse-transcription polymerase chain reaction (qRT-PCR). The abundance of the gene transcripts was calculated relative to endogenous control (β-tubulin gene) using the 2^–ΔΔCT^ method. Error bars represent the standard deviation.

Based on previous studies of methionine biosynthesis in yeasts and filamentous fungi, an outline of predicted methionine metabolism pathway in *M. oryzae* was described, as shown in Figure 9A. Cysteine is converted to methionine by a pathway that begins with cystathionine γ-synthase and cystathionine β-lyase, respectively (Figure 9A). Methionine can then be generated from homocysteine, which is methylated by 5-methyltetrahydrofolate via methionine synthase (cobalamin- independent methionine synthase). Several genes were predicted to be involved in this process and are listed in Figure 9A. To determine the expression patterns of genes putatively involved in methionine metabolism, qRT-PCR assays were performed. Deletion of *MET12* resulted in down-regulation of *MS1* (MGG_06712), *SHM1* (MGG_13781) and *HCS1* (MGG_07195), which putatively encode methionine synthase, serine hydroxymethyltransferase and homocysteine synthase, respectively, while all other genes were up-regulated (Figure 9B). However, deletion of *MET13* led to 2.19-14.83 fold up-regulation of most genes, although *MS1* was down-regulated (Figure 9B). Interestingly, most of the genes involved in methionine metabolism were significantly down-regulated in the *Δmet13Δmet12* mutant (Figure 9B). These results indicate that both *MET13* and *MET12* play important roles in regulating expression of genes encoding enzymes involved in methionine metabolism in *M. oryzae*.

Furthermore, we selected several sulfur metabolism genes (MGG_10308 and MGG_07920 putatively encoding arylsulfatase, MGG_06466 encoding cysteine synthase and MGG_07195 encoding homocysteine synthase by *M. oryzae*) and carried out qRT-PCR analysis of their expression in *Δmet13* and *Δmet12* mutants. The results showed that MGG_10308, MGG_07920, MGG_06466 and MGG_07195 were slightly up-regulated by 1.27±0.2, 1.12±0.25, 1.24±0.36 and 1.31±0.18 fold in the *Δmet12* mutant, respectively, and 1.25±0.11, 1.42±0.12, 1.27±0.39 and 1.23±0.24 fold in the *Δmet13* mutant, respectively. These data suggested that above sulfur metabolism genes were not significantly regulated by the deletion of *MET12* or *MET13*.

## Discussion

MTHFR is a critical enzyme for converting 5,10-CH_2_-THF to 5-CH_3_-THF, and the latter then serves as a major methyl donor in the re-methylation of homocysteine to methionine. Most fungal genomes, including yeasts and filamentous fungi, contain two types of MTHFRs [1,6-8]. In this report, we identified a non-pathogenic mutant WH672 of *M. oryzae* using T-DNA insertional mutagenesis, and identified *MET13*, which putatively encodes a homologue of *S. cerevisiae* Met13 (MTHFR1). We also identified *MET12*, which putatively encoding a homologue of *S. cerevisiae* Met12 (MTHFR2). Deletion of *MET13* resulted in methionine auxotrophy and deletion of *MET12* also caused slow growth on MM medium without methionine, indicating that both genes are required for methionine synthesis by *M. oryzae*. Phenotypic analysis showed that *MET13* was essential for mycelium pigment, conidiation, appressorium formation and virulence by *M. oryzae*. However, deleting *MET12* led only to minor phenotypic alterations, such as a reduction in sporulation. These results suggest that MTHFR1 may provide the majority of cellular MTHFR activity, although MTHFR2 is also involved in methionine synthesis in *M. oryzae*.

In *S. cerevisiae*, deletion of *MET13* but not *MET12* leads to methionine auxotrophy and results in loss of MTHFR activity, although Met12 is also responsible for approximately 15% MTHFR activity [1]. Similarly, in *Schizosaccharomyces pombe*, analysis of MTHFR activity shows that Met9 (a Met13 homologue) is responsible for approximately 80 to 85% of the total MTHFR activity [6]. In *A. nidulans*, disruption of either *METF* (a *MET13* homologue) or *META* (a *MET12* homologue) results in methionine auxotrophy [7]. Analysis of enzymatic activity shows that the META-encoded enzyme is responsible for only 10-15% of total MTHFR activity [7]. Unlike *metA* strains, *metF* disruptant strains grow poorly on minimal medium supplemented with 0.25 mM methionine and required at least 1 mM methionine for growth and conidiation [7]. Methionine auxotrophy of *metA* but not *metF* mutants can be overcome by the addition of homocysteine [7]. In *F. graminearum*, deletion of either of the two MTHFR encoding genes, *FgMET13* and *FgMET12*, leads to methionine auxotrophy [8]. However, unlike in *A. nidulans*, homocysteine is unable to rescue either of the gene deletion mutants. These previous studies suggest that MTHFR1 and MTHFR2 may have overlapping catalytic properties, although MTHFR1 appears to be more important to cellular MTHFR activity. In this study, methionine but not homocysteine could complement methionine auxotrophy of *Δmet13* mutants (Figure 5). However, growth defects of *Δmet12* mutants were rescued by exogenous methionine and also partially overcome by adding homocysteine, suggesting that regulation in *M. oryzae* is similar to *A. nidulans* but distinct to *F. graminearum*. Additionally, the double deletion mutants of *MET13* and *MET12* were also methionine auxotrophs and had similar phenotypes to *Δmet13* mutants, suggesting that Met13 might be the predominant source of methionine biosynthetic activity in *M. oryzae*.

In *Leishmania major* (a protozoan parasite), a causative agent of human diseases, *LmMTHFR* null mutants cannot utilize exogenous homocysteine for growth and grow poorly under conditions of methionine limitation, but are still virulent in a mouse model of infection [36]. To date, MTHFR has not been reported as a virulent determinant in phytopathogenic fungi. Recently, it has been reported that MTHFR is involved in the plasma membrane redox system required for pigment biosynthesis in filamentous fungi [8,37]. In *F. pseudograminearum*, an exogenous T-DNA integration in the promoter of *FpsMET13* (a *MET13* homologue) caused accumulation of yellow pigment instead of aurofusarin in mycelia [8,37]. In *F. graminearum*, either *FgMET13* or *FgMET12* is required for pigment biosynthesis [8]. Deletion of *FgMET12* displayed a delay in the production of the mycelium pigment aurofusarin and instead accumulated norrubrofusarin and rubrofusarin, while the defects could be rescued by high concentration of exogenous methionine or prolonged incubation [8]. The *Δfgmet13* mutants, however, remained aurofusarin deficient at all tested methionine concentrations and instead accumulated nor-rubrofusarin and rubrofusarin [8]. Here, we showed that deletion of *M. oryzae MET13* led a defect in plant infection-related morphogenesis and pathogenicity (Figures 2 and 3). The *Δmet13* mutants were completely unable to produce conidia, form appressoria or cause blast disease. In contrast to *MET13*, deletion of *MET12* led to a significant reduction in conidiation on CM medium (Figure 3B and C), but *Δmet12* mutants were still able to form melanized appressorium and cause blast disease (Figure 2B and C; Figure 3D). Similar to *F. graminearum*, colony pigmentation of *Δmet13* mutants was significantly reduced compared with *Δmet12* mutants (Figure 2A), suggesting that Met13 is also involved in pigment synthesis in *M. oryzae*.

In *S. cerevisiae*, overexpression of *MET12* failed to rescue methionine auxotrophy of a *MET13* deletion strain [1]. In this study, qRT-PCR analysis showed that deletion of *MET13* led to a significant increase in *MET12* expression and vice versa (Figure 8), indicating that the *MET12* expression in a *Δmet13* mutant is insufficient to complement defects caused by deletion of *MET13*. Similarly, overexpression of *MET12* in a *Δmet13* mutant did not overcome its mutant phenotypes.

Surprisingly, deletion of *MET13* (but not *MET12*) led to significant up-regulated expression of several genes involved in methionine metabolism such as *SHM1* and *SAM1* encoding serine hydroxymethyltransferase and S-adenosyl-methionine synthase, respectively (Figure 9B). Biosynthesis of methionine was significantly impaired in *Δmet13* mutants, but the mutants may have the ability to synthesize some methionine by up-regulated expression of *MET12* and other genes, including *SHM1* and *SHM2*. In addition, since S-adenosyl methionine (SAM) is an important substrate involved in methyl group transfers, the *Δmet13* mutants may up-regulate the expression of *SAM1* to maintain the normal levels of SAM in cells. However, the exact regulation mechanism of these genes involved in methionine metabolism still remains to be elucidated.

Methionine is a crucial sulfur-containing amino acid, which is not only an essential component of proteins but also acts in the initiation of translation. Previous studies showed that the methionine synthesis pathway may be involved in virulence in phytopathogenic fungi. In *F. graminearum*, deletion of either *CBL1* or *MSY1*, encoding cystathionine beta-lyase and methionine synthase, respectively, were methionine auxotrophs and significantly reduced in virulence on corn silks and wheat heads [38]. Recently, it was shown that *FgMETB*, encoding cystathionine gamma-synthase, is essential for methionine biosynthesis in *F. graminearum* [39]. Deletion of *FgMETB* led to a significant reduction of conidial germination in 2% sucrose solution and virulence on wheat heads [39]. In *M. oryzae*, it has been reported that a methionine auxotrophic mutant, 130, was reduced in virulence on rice seedlings [40]. Additionally, *M. oryzae CBS1* encoding cystathionine β-synthase (CBS) which catalyzes formation of cystathionine from homocysteine and serine has been functionally characterized [41]. However, the null (*cbs1*) mutants were still virulent on rice [41]. *M. oryzae STR3* encodes cystathionine β-lyase that converts cystathionine to homocysteine during the biosynthesis of methionine. A recent report showed that deletion of *STR3* resulted in methionine auxotrophy and methionine-requiring phenotypes could be complemented by supplementation with methionine [42]. Live-cell-imaging showed that the *Δstr3* mutants failed to develop in host cells and were unable to cause blast disease, although they could produce normal appressoria and enter host cells, indicating some nutrients might not be readily available to *M. oryzae* in rice host cells [42]. Our results demonstrated that *Δmet13* mutants were strict methionine auxotrophs and essential for infection-related development and initial infection of both rice and barley cells. This suggests that methionine is limiting under these conditions and its presence is essential for development of mature appressoria which require melanin synthesis and cell wall differentiation to be functional. When considered together our results are consistent with *MET13* encoding the major MTHFR activity in *M. oryzae* which plays a significant role in ensuring efficient methionine biosynthesis during the starvation conditions associated with pre-penetration development of appressoria from spores and during initial leaf tissue colonization. They also point to methionine being a critical amino acid during development of spores, perithecia and appressoria of the rice blast fungus. Methionine biosynthesis may therefore be valuable as a potential fungicide target given its pivotal nature in the initiation of plant infection.

## Materials and Methods

### Strains and growth conditions

All mutants described in the present study were generated from *M. oryzae* wild-type strain Guy11 (Table S1). Standard growth and storage procedures for fungal strains were performed, as described previously [32]. *Escherichia coli* strain DH-5α was used for routine bacterial transformations and maintenance of plasmids in this study.

### Fungal growth, sporulation, appressorium formation and genetic crosses

Vegetative growth was assessed by measurement of colony diameter in plate cultures of *M. oryzae* grown on CM medium at 25°C for 10 d. Conidial development was assessed by harvesting conidia from the surface of 12-day-old plate cultures and determining the concentration of the resulting conidial suspension using a haemocytometer (Corning). Appressorium formation and penetration were measured on a hydrophobic coverslips and onion epidermis surfaces at 25°C for 24 h, respectively. The percentage of conidia formed appressoria or appressoria formed penetration pegs were determined by microscopic examination of at least 100 conidia or appressoria. Fertility assays were carried out by pairing Guy11 (*MAT1-2*) and other mutants with standard tester strain TH3 (*MAT1-1*) on oatmeal agar (OMA) plates, as described previously [28,29]. The junctions between the mated individuals were examined for the capacity to form perithecia. Each test was repeated at least three times.

### Pathogenicity assays

Both cut-leaf and spraying methods were used for plant infection assays as described previously [30]. For cut-leaf assays, the mycelium plugs from 10-day old CM cultures were used to place onto leaf fragments of susceptible hosts: barley cv Golden Promise and rice cv CO-39. For rice seedling infection assays, conidia were harvested from the CM cultures with 1 mM methionine and conidial suspensions were diluted in 0.2% gelatin with 1 mM methionine solution to 1 × 10^5^ conidia ml^-1^. The conidial suspensions were then used to spray seedlings of CO39. Plants were incubated for 5 days for disease symptom development.

### Construction of vectors and fungal transformation

For the construction of the *MET13* gene replacement vector, a 1.4 kb *HPH* gene cassette, which encodes hygromycin phosphotransferase under control of the *A. nidulans* TrpC promoter [43], was amplified with primers HPH-BF and HPH-BR (Table S2) using pCB1003 as a template. 1.1 kb flanking sequences on either border of the *MET13* gene locus were amplified using primer pairs of WF1/WR2 and WF3/WR4 and cloned sequentially into pGEM-T easy vectors (Promega, Madison, WI, U.S.A.) to generate pGEM-13L and pGEM-13R, respectively. pGEM-13R was digested with *Bam*HI and *Spe*I and the releasing fragment was inserted into pGEM-13L with the correspondent ends, leaving a single *Bam*HI site for insertion of the *HPH* cassette with *Bam*HI ends. The resulting construct vector, pMET13-KO, was linearized and transformed into *M. oryzae* Guy11 for generating homologous recombinants.

A similar strategy was used for the construction of the *MET12* gene replacement vector, but the *HPH* gene was substituted with a *BAR* gene for transformant selection. A 0.94 kb *BAR* gene cassette, which encodes phosphinothricin acetyl transferase under control of the *A. nidulans* TrpC promoter, was amplified with primers Bar-KF and Bar-KR (Table S2) using pMLH21-bar [44] as a template, and cloned sequentially into pGEM-T easy vector to generate pGEM-KB. 1.1 kb flanking sequences on either border of the *MET12* gene locus were amplified using primer pairs of WF5/WR6 and WF7/WR8 and cloned sequentially into pGEM-T easy vectors to generate pGEM-12L and pGEM-12R, respectively. pGEM-12R was digested with *Kpn*I and *Sac*I and the releasing fragment was inserted into pGEM-12L with the correspondent ends, leaving a single *Kpn*I site for insertion of the *BAR* gene cassette with *Kpn*I ends from pGEM-KB. The resulting construct vector, pMET12-KO, was linearized and transformed into *M. oryzae* Guy11 for generating homologous recombinants.

The *MET13* complementation vector (C-terminal GFP tagging vector), pMET13-GFP, was constructed by amplification of a 3.3 kb fragment including the 2.0 kb *MET13* gene-coding sequence and a 1.3 kb promoter region using primers 13F and 13R (Table S2) and by amplification of 1.5 kb fragment GFP allele [45] carrying the *A. nidulans* trpC terminator using primers GFP-13F and GFP-13R (Table S2). Construction of pMET13-GFP was carried out by cloning the 3.3 kb PCR product into pGEM-T easy vector to generate pGM-M13. The 1.5 kb GFP allele was then cloned to pGEM-T easy vector and digested with *Hin*dIII to release the GFP allele with *Hin*dIII ends, which was inserted at the *Hin*dIII site of pGM-M13 to create pGM-MET13-GFP. Finally, pGM-MET13-GFP was digested with *Eco*RI to release the *MET13* C-terminal GFP tagging fragment, which was inserted into the *Eco*RI site of pCB1532, which contains the *ILV1* allele conferring resistance to sulfonylurea [46] to give pMET13-GFP. The resulting plasmid was transformed into the *Δmet13* mutant

(K56).

Double knockout *Δmet13Δmet12* mutants were created by transformation of pMET13-KO into strain K12-7 (*△met12*) and were confirmed by PCR amplification (Figure S3A and F).

### Nucleic acid manipulation, Southern blot analysis and quantitative RT-PCR

DNA extraction was performed as described previously [32], while general procedures for nucleic acid manipulation followed standard protocols [47]. Southern blot analysis was performed by the digoxigenin (DIG) high prime DNA labeling and detection starter Kit I (Roche, Mannheim, Germany). For quantitative RT-PCR, total RNA extraction, synthesis of the first strand cDNA, PCR amplification and relative gene expression were performed and calculated as described previously [28,30,48]. The primer pairs of RT-1F/ RT-2R and RT-3F/ RT-4R (Table S2) were used to determine the relative expression of *MET13* and *MET12*, respectively. Expressions of the genes involved in methionine metabolism were analyzed with the corresponding primers listed in Table S2. *M. oryzae* beta-tubulin gene (MGG_00604.6) amplified with the primer pairs of BT-F/BT-R was used as an endogenous control. All qRT-PCR reactions were repeated three times.

## Supporting Information

Figure S1 **Amino acid sequence alignment between Met13 and Met12 of *Magnaporthe oryzae*.** Alignment of the predicted amino acid sequence between Met13 and Met12. Identical amino acids are shown on a black background and similar amino acids are shown on a light gray background.
(RTF)

Figure S2 **Phylogenetic analysis of *Magnaporthe oryzae* MTHFR1 (Met13) and MTHFR2 (Met12) with the homologues from other fungal species.** Phylogenetic tree was constructed by observed divergency distance method in the program DNAMAN. Numbers at the nodes in the rooted tree represent bootstrapping value on 1000 replications. Abbreviations and numbers correspond to species names and GenBank accession numbers, respectively. Ac, *Aspergillus clavatus*; Af, *A. fumigatus*; An, *A. nidulans*; Ang, *A. niger*; Ao, *A. oryzae*; Ca, *Candida albicans*; Cg, *Chaetomium globosum*; Gg, *Glomerella graminicola*; Gz, *Gibberella zeae*; Mo, *Magnaporthe oryzae*; Nc, *Neurospora crassa*; Nh, *Nectria haematococca*; Pa, *Podospora anserine*; Pc, *Penicillium chrysogenum*; Pm, P*. marneffei*; Sc, *Saccharomyces cerevisiae*; Ss, *Sclerotinia sclerotiorum*. The bar indicates 0.05 distance units. DNAMAN version 5.2.2 program was used for alignment and phylogenetic tree constrution.
(DOC)

Figure S3 **Targeted gene replacement of *MET13* and *MET12*.** (**A**) Construction of the vector pMET13-KO and targeted gene replacement of *MET13*. (**B**) Southern blot analysis. Genomic DNA was digested with *Kpn*I and probed with a 0.8 kb fragment amplified with the primers P13F and P13R. Lane 1 to lane 5: ECT5 (ectopic), K56 (*Δmet13*), Guy11 (the wild-type strain), K73 (*Δmet13*) and ECT8 (ectopic). (**C**) PCR amplification with the primers F13 and R13. (**D**) Construction of the vector pMET12-KO and targeted gene replacement of *MET12*. (**E**) Southern blot analysis. Genomic DNA was digested with *Nde*I and probed with a 1.0 kb fragment amplified with the primers P12F and P12R. Lane 1 to 4: K12-13 (*Δmet12*), K12-7 (*Δmet12*), E12-8 (ectopic) and Guy11 (**F**) *Δmet13Δmet12* mutants confirmed by PCR analysis. PCR amplification with the primers F13 and R13 (top panel) or RT-1F and DK-R (bottom panel). Lane 1 to 7: Guy11, DK7 (*Δmet13Δmet12*), Guy11, DK13 (*Δmet13Δmet12*), DK18 (*Δmet13Δmet12*), DE-1 and DE-2 (ectopic). B = *Bam*HI; K = *Kpn*I; N = *Nde*I; S=*Sac*I; Sp = SpeI. Asterisk represents restriction sites introduced or derived from vectors (double asterisks).
(TIF)

Figure S4 **Fertility assay of the wild-type strain Guy11, *Δmet13*, *Δmet13MET13*, *Δmet12* and *Δmet13Δmet12* on OMA medium.** The crosses of Guy11 × TH3 and C3 (*Δmet13MET13*) × TH3 formed numerous perithecia, asci and ascospores on oatmeal medium (OMA), while K12-7 (*Δmet12*) × TH3 formed less perithecia, asci and ascospores. No perithecia was observed for the crosses of K56 (*Δmet13*) × TH3 and DK7 (*Δmet13Δmet12*) × TH3.
(TIF)

Figure S5 **Growth patterns and conidiation of the *Δmet13* mutant on CM medium with methionine.** (**A**) Aerial growth and colony pigment of the *Δmet13* mutant (K56) were complemented by the supplement of methionine. The photographs were taken from 10 day-old cultures on CM at 25°C. (**B**) Bar chart showing the conidial production. The ability to produce conidia of the *Δmet13* mutant was partially restored by adding exogenous methionine. Error bars represent standard deviation. Concentrations of methionine were: 0.5, 1, 2, 4, 6, 8 and 10 mM. Lower case letters indicate significant differences at *P* = 0.05.
(TIF)

Figure S6 **Growth patterns of the wild-type and other strains in liquid CM medium with or without methionine.** Mycelium growth patterns of the strains in liquid CM medium at 25°C for 48 h (bottom). Growth defects of *MET13* gene deletion or disruption mutants in liquid CM medium were complemented by the supplement of exogenous methionine. Guy11, the wild-type strain; *Δmet13*, K56; *Δmet12*, K12-7; *Δmet13MET13*, C3; WH672, the T-DNA insertional mutant; *Δmet13Δmet12*, DK7. Met: 1 mM methionine; Scale bars = 5 mm.
(TIF)

Table S1 **Wild-type and recombinant strains of *Magnaporthe oryzae* used in this study.**
(DOC)

Table S2 **PCR primers used in this study.**
(DOC)
