# Supplementary material for: The MET13 Methylenetetrahydrofolate Reductase Gene Is Essential for Infection-Related Morphogenesis in the Rice Blast Fungus Magnaporthe oryzae
Source: PLoS One. 2013 Oct 7;8(10):e76914. doi: 10.1371/journal.pone.0076914 (PMC3792160; doi:10.1371/journal.pone.0076914)
Supplement: Figure S1 — Amino acid sequence alignment between Met13 and Met12 of Magnaporthe oryzae. Alignment of the predicted amino acid sequence between Met13 and Met12. Identical amino acids are shown on a black background and similar amino acids are shown on a light gray background. (RTF) [file pone.0076914.s001.rtf]

Met13    1 MLAETEKTGKPS-----FSFEYFPPKTAQGVQNLYDRMERMYHFG-PKFIDITWGAGGRI
Met12    1 MDKITDKIAALPPDASYFSLEFFPPKTAMGFSNLRDRLDRMARALRPLFVNVTWGAGGST


Met13   55 AELTCEMVTQAQTYFGLETCMHLTCTDMGVEKVNDALSKAYKAGCTNILALRGDPPRD--
Met12   61 AQKSLELAEICQRELALTTCLHLTCTNMSRKLIDKALEDAKALGIRNILALRGDPPRPGE


Met13  113 ---KEKWEAAQDGFNYAKDLVSHIRKTYGDHFDIGVAGYPEG-----CDDNKDEDLLLDH
Met12  121 YAIPDDSEGDVNEFTWAIDLVRYIKLNHGDYFCVGVAAYPEGHAEESHPTNQSLEHDLPY


Met13  165 LKEKVDMGASFIVTQMFYDADNFVRWVGRVRERG----ITVPIIPGIMPIATYASFLRRA
Met12  181 LVEKTQAGADFIITQLFFDISAYEKFEKTLREHPSGAFKDIPIIPGLMPIQNYQMIKRTT


Met13  221 NHMQARIPEEWLQRLEPIKTDDAAVRIVGRQLVVELCR-----KILAAGIHHLHFYTMNL
Met12  241 KLSHAKIPDPLMARLDAVKKDDEQVKKVGVDIISELVDQVKEVKNRSSGPKGFHFYTLNL


Met13  276 AQSTALILEDLDWLPS----------------PNKPLKHALPWKQSLGLGRREEDVRPIF
Met12  301 EKAVSFIVERTNLIPATTPDDDEVAVVDDVALPSIHLNGATPAKDHLSVSSLHPPTHTNS


Met13  320 WR------------------------------------------------NRNKSYVMRT
Met12  361 RRPSTIGSDPRNRVIVSSGRPASHPDYEATGFEASVPAQAINSRANTLAISEGEGVLGRE


Met13  332 QDWDEFPNGRWGDSRSPAFGELDAYGIGLLGTNEQNRKKFGEPKSVKDIATLFVRYVQKE
Met12  421 ATWDDYPNGRWGDARSPAYGEIDGYGVSLHMSGTQAVQLWGRPATVEDVSNIFMRHLRGA


Met13  392 VDTLPWSESPLDAEAEQIRDDLIDLNLRGLITINSQPAVNGVRSTHPIHGWGPPNGYVYQ
Met12  481 LPAIPWSEEEFNAETETIRDTLLALNSRGWWTVASQPAVNGLRSNDRTFGWGPQNGFVFQ


Met13  452 KAYLELLVHPAIFEQLKERIHDHPDLTYYAVTKSGNLHTNATYEG---------------
Met12  541 KAFVEMFLPSKDWKALREKLLSEKDVVCFYASNAKGDFESSDGGGDAPDGAPTGNGFADE


Met13  497 --------PNAVTWGVFPGKEIVQPTVVENISFLAWKDEAFQLGMEWARCHDQNTPSRIL
Met12  601 QPLPSGSSTNAVTWGVFPGKEIVTPTIIEEVSFRAWSEEAFGIWAEWAKVYGKDSPSEKL


Met13  549 IQSMMQEWYLVNIVNNDFHAPRTIFDTLKGLTVPDIDTEIVPPALPTAVPEAAAINGERN
Met12  661 LESLRADLWLVNIIHHDYIEKEALWDLLLK------------------------------


Met13  609 GERNGAQANGATAAVTAS
Met12      ------------------


                                                   Fig.S1
