# Supplementary material for: The MET13 Methylenetetrahydrofolate Reductase Gene Is Essential for Infection-Related Morphogenesis in the Rice Blast Fungus Magnaporthe oryzae
Source: PLoS One. 2013 Oct 7;8(10):e76914. doi: 10.1371/journal.pone.0076914 (PMC3792160; doi:10.1371/journal.pone.0076914)
Supplement: Figure S2 — Phylogenetic analysis of Magnaporthe oryzae MTHFR1 (Met13) and MTHFR2 (Met12) with the homologues from other fungal species. Phylogenetic tree was constructed by observed divergency distance method in the program DNAMAN. Numbers at the nodes in the rooted tree represent bootstrapping value on 1000 replications. Abbreviations and numbers correspond to species names and GenBank accession numbers, respectively. Ac, Aspergillus clavatus; Af, A. fumigatus; An, A. nidulans; Ang, A. niger; Ao, A. oryzae; Ca, Candida albicans; Cg, Chaetomium globosum; Gg, Glomerella graminicola; Gz, Gibberella zeae; Mo, Magnaporthe oryzae; Nc, Neurospora crassa; Nh, Nectria haematococca; Pa, Podospora anserine; Pc, Penicillium chrysogenum; Pm, P. marneffei; Sc, Saccharomyces cerevisiae; Ss, Sclerotinia sclerotiorum. The bar indicates 0.05 distance units. DNAMAN version 5.2.2 program was used for alignment and phylogenetic tree constrution. (DOC) [file pone.0076914.s002.doc]

Ac_XP_001275420

Af_EDP54646

100

Ao_XP_001821959

80

Ang_XP_001399463

An_XP_663487

Pc_XP_002560275

96

Pm_XP_002152051

100

**Mo_MGG_01728**

Cg_XP_001223823

Pa_XP_001910226

82

Nc_XP_961729

96

98

Gg_EFQ33219

77

Gz_XP_389748

Nh_XP_003046086

100

100

Ss_XP_001589131

100

99

Ca_XP_720117

Sc_EGA62396

100

100

Ss_XP_001597813

Cg_XP_001229015

Pa_XP_001911720

80

Nc_XP_958764

99

**Mo_MGG_08171**

Gz_XP_387303

Nh_XP_003050376

100

Gg_EFQ27891

98

100

An_XP_681484

Ang_XP_001393897

Ao_BAE61576

AC_XP_001276185

Af_EDP51195

99

77

Pc_XP_002568600

100

Pm_XP_002147724

100

100

Ca_EEQ47532

Sc_NP_015302

93

100

0.05

**MTHFR1**

**MTHFR2**

**Fig. S2**
