# Supplementary figures and images for: The MET13 Methylenetetrahydrofolate Reductase Gene Is Essential for Infection-Related Morphogenesis in the Rice Blast Fungus Magnaporthe oryzae
Source: PLoS One. 2013 Oct 7;8(10):e76914. doi: 10.1371/journal.pone.0076914 (PMC3792160; doi:10.1371/journal.pone.0076914)

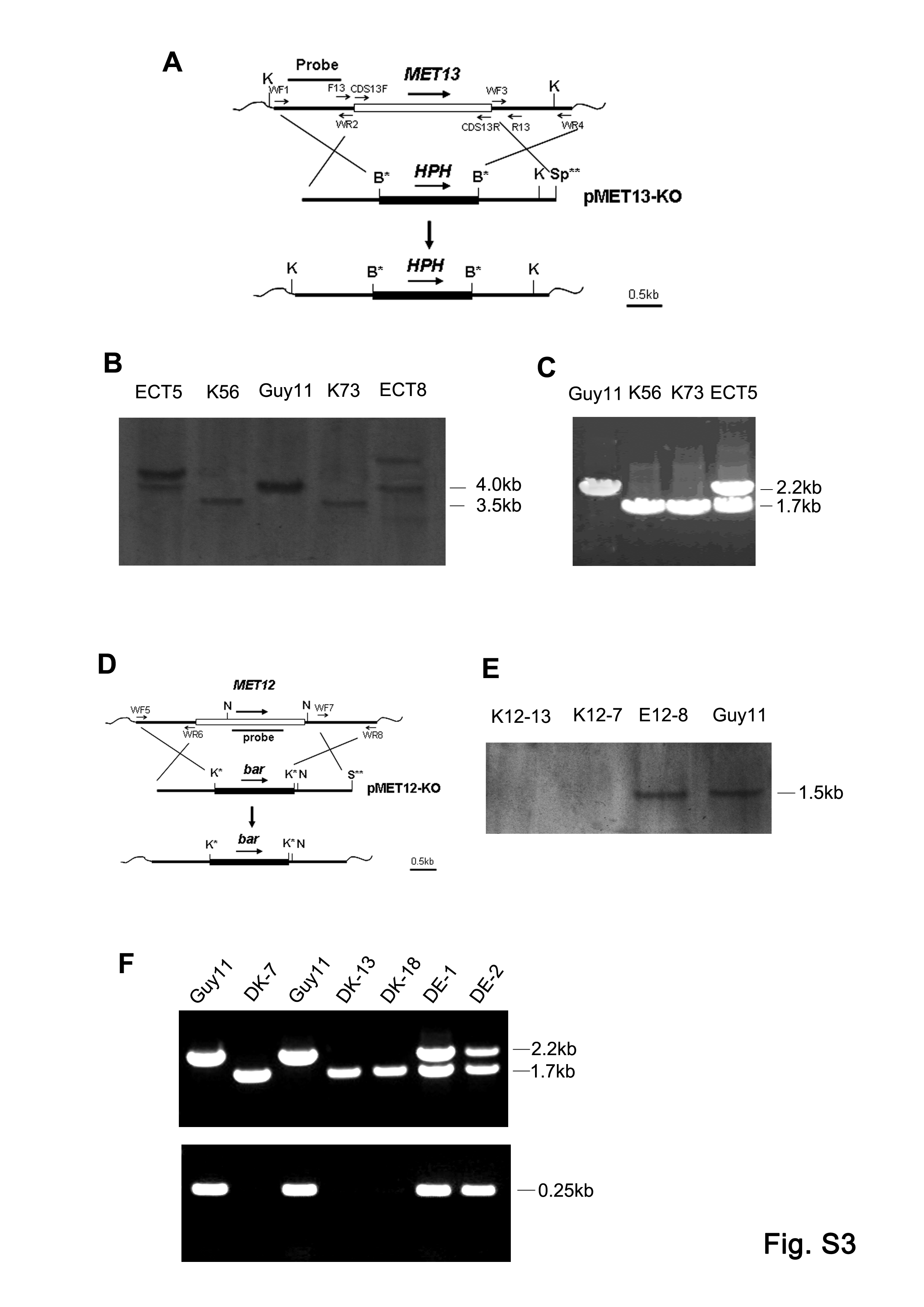

Supplement: Figure S3 — Targeted gene replacement of MET13 and MET12. (A) Construction of the vector pMET13-KO and targeted gene replacement of MET13. (B) Southern blot analysis. Genomic DNA was digested with KpnI and probed with a 0.8 kb fragment amplified with the primers P13F and P13R. Lane 1 to lane 5: ECT5 (ectopic), K56 (Δmet13), Guy11 (the wild-type strain), K73 (Δmet13) and ECT8 (ectopic). (C) PCR amplification with the primers F13 and R13. (D) Construction of the vector pMET12-KO and targeted gene replacement of MET12. (E) Southern blot analysis. Genomic DNA was digested with NdeI and probed with a 1.0 kb fragment amplified with the primers P12F and P12R. Lane 1 to 4: K12-13 (Δmet12), K12-7 (Δmet12), E12-8 (ectopic) and Guy11 (F) Δmet13Δmet12 mutants confirmed by PCR analysis. PCR amplification with the primers F13 and R13 (top panel) or RT-1F and DK-R (bottom panel). Lane 1 to 7: Guy11, DK7 (Δmet13Δmet12), Guy11, DK13 (Δmet13Δmet12), DK18 (Δmet13Δmet12), DE-1 and DE-2 (ectopic). B = BamHI; K = KpnI; N = NdeI; S=SacI; Sp = SpeI. Asterisk represents restriction sites introduced or derived from vectors (double asterisks). (TIF) [file pone.0076914.s003.tif]

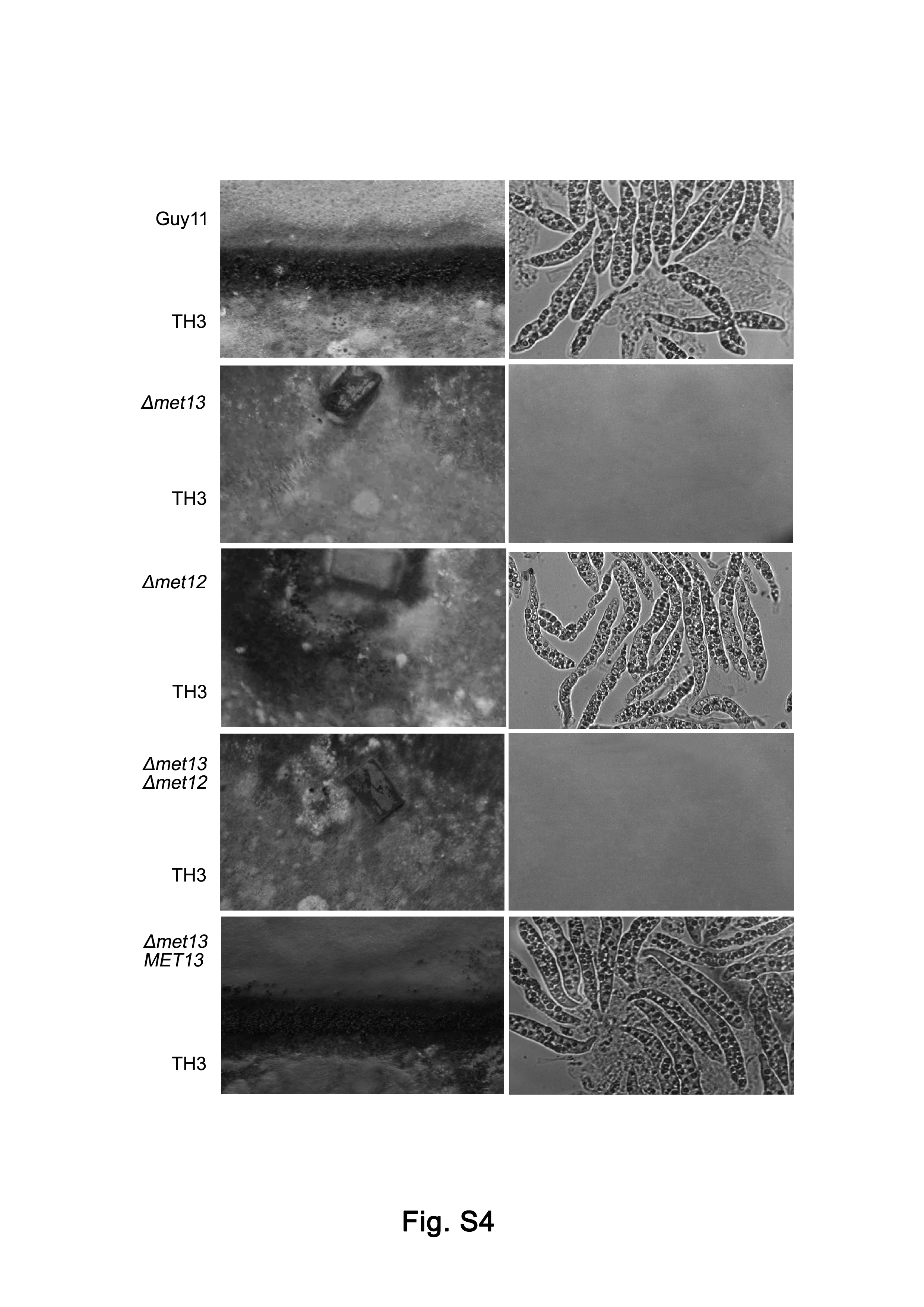

Supplement: Figure S4 — Fertility assay of the wild-type strain Guy11, Δmet13, Δmet13MET13, Δmet12 and Δmet13Δmet12 on OMA medium. The crosses of Guy11 × TH3 and C3 (Δmet13MET13) × TH3 formed numerous perithecia, asci and ascospores on oatmeal medium (OMA), while K12-7 (Δmet12) × TH3 formed less perithecia, asci and ascospores. No perithecia was observed for the crosses of K56 (Δmet13) × TH3 and DK7 (Δmet13Δmet12) × TH3. (TIF) [file pone.0076914.s004.tif]

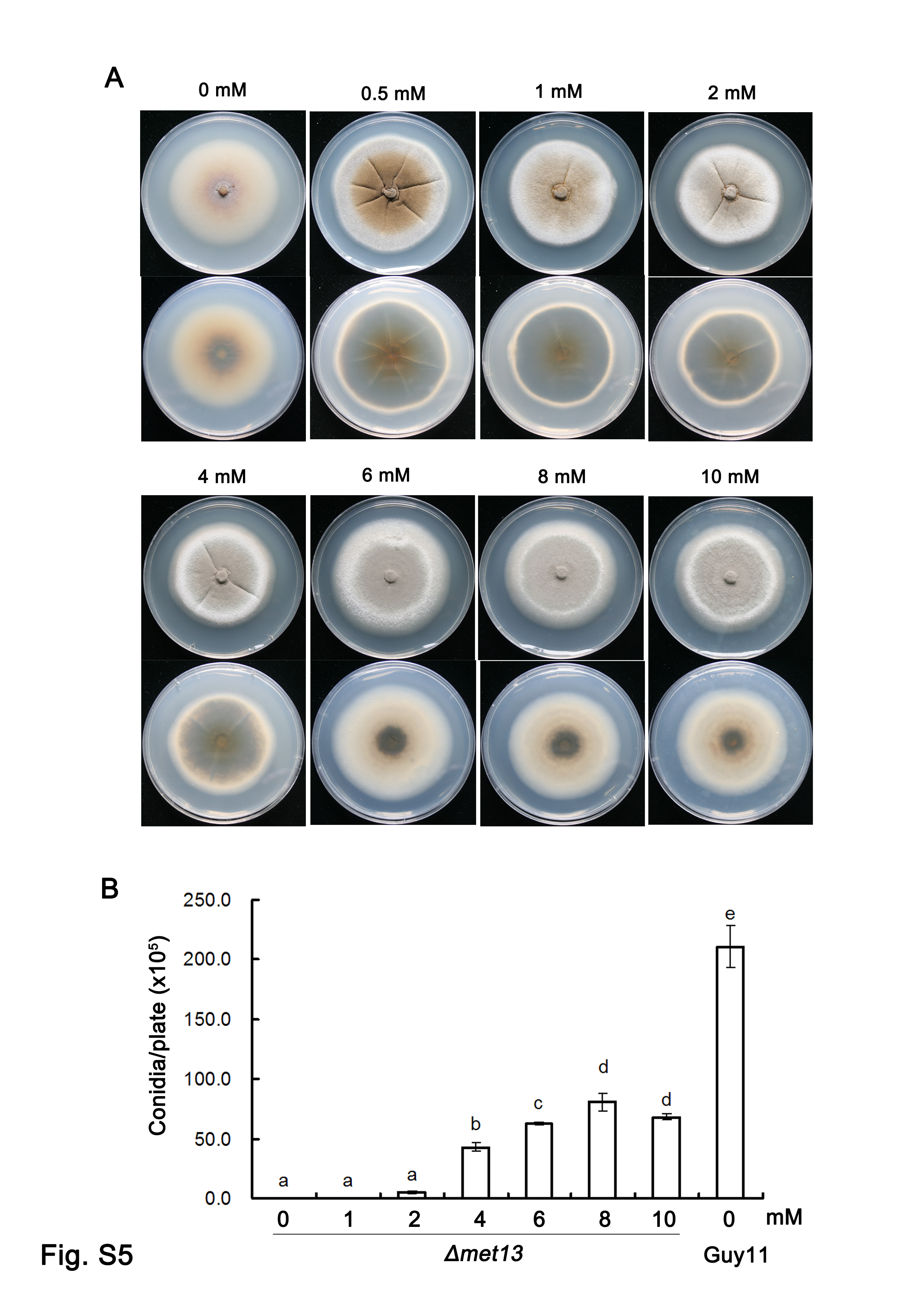

Supplement: Figure S5 — Growth patterns and conidiation of the Δmet13 mutant on CM medium with methionine. (A) Aerial growth and colony pigment of the Δmet13 mutant (K56) were complemented by the supplement of methionine. The photographs were taken from 10 day-old cultures on CM at 25°C. (B) Bar chart showing the conidial production. The ability to produce conidia of the Δmet13 mutant was partially restored by adding exogenous methionine. Error bars represent standard deviation. Concentrations of methionine were: 0.5, 1, 2, 4, 6, 8 and 10 mM. Lower case letters indicate significant differences at P = 0.05. (TIF) [file pone.0076914.s005.tif]

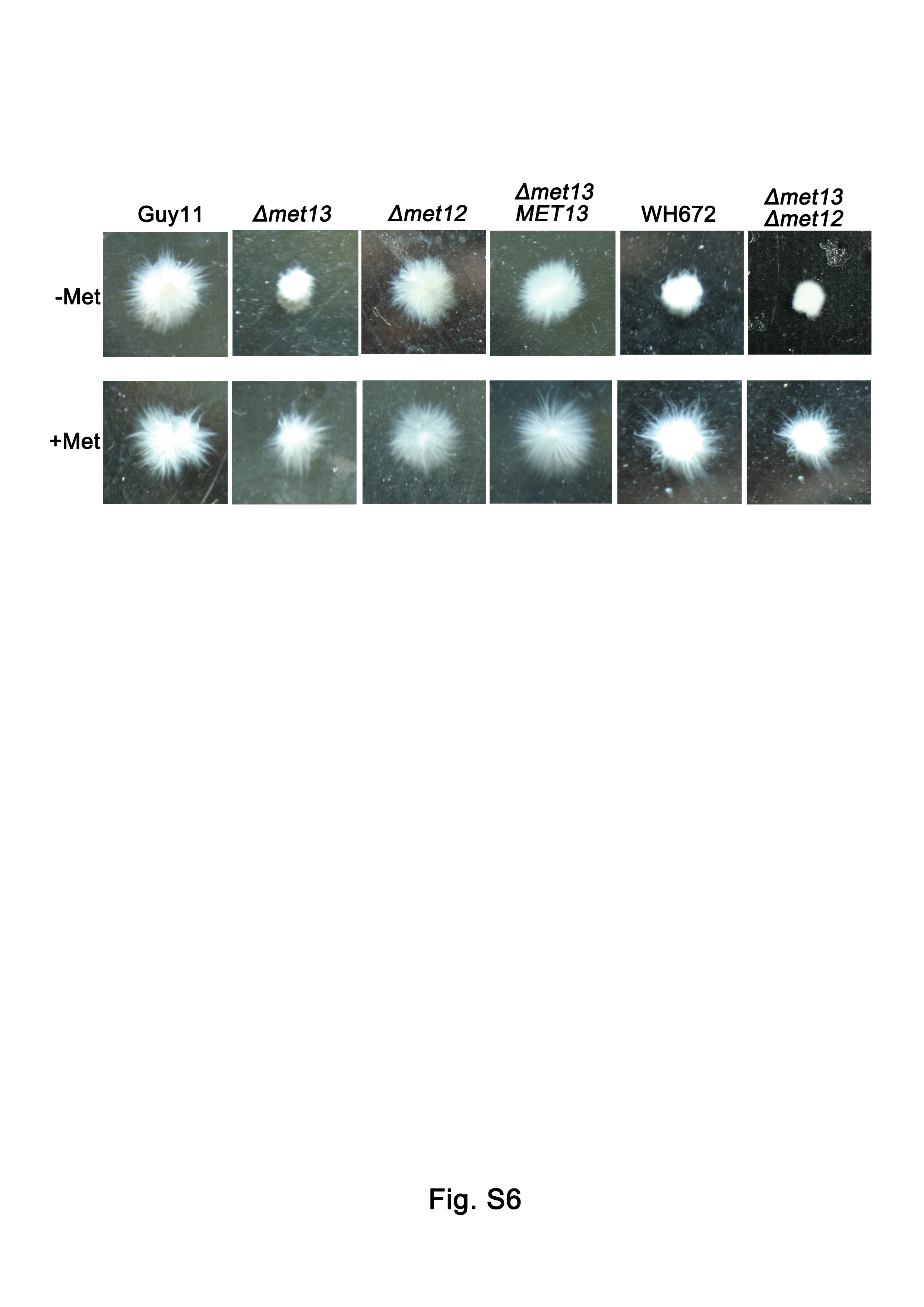

Supplement: Figure S6 — Growth patterns of the wild-type and other strains in liquid CM medium with or without methionine. Mycelium growth patterns of the strains in liquid CM medium at 25°C for 48 h (bottom). Growth defects of MET13 gene deletion or disruption mutants in liquid CM medium were complemented by the supplement of exogenous methionine. Guy11, the wild-type strain; Δmet13, K56; Δmet12, K12-7; Δmet13MET13, C3; WH672, the T-DNA insertional mutant; Δmet13Δmet12, DK7. Met: 1 mM methionine; Scale bars = 5 mm. (TIF) [file pone.0076914.s006.tif]
