# Supplementary material for: The MET13 Methylenetetrahydrofolate Reductase Gene Is Essential for Infection-Related Morphogenesis in the Rice Blast Fungus Magnaporthe oryzae
Source: PLoS One. 2013 Oct 7;8(10):e76914. doi: 10.1371/journal.pone.0076914 (PMC3792160; doi:10.1371/journal.pone.0076914)
Supplement: Table S1 — Wild-type and recombinant strains of Magnaporthe oryzae used in this study. (DOC) [file pone.0076914.s007.doc]

**Table 1. Wild-type and recombinant strains of *Magnaporthe oryzae* used in this study**

| **Strain** | **Brief description** | **Reference** |
| --- | --- | --- |
| Guy11 | Wild-type, MAT1-2 | [31] |
| TH3 | Wild-type, MAT1-1 | [35] |
| WH672 | T-DNA insertional mutant | This study |
| K56, K73, K85, K96 | *△met13* mutants of Guy11 | This study |
| ECT5，ECT8 | Ectopic transformants of Guy11 with integration of pMET13-KO | This study |
| C3, C5, C6 | *△met13* transformed with pMET13-GFP | This study |
| K12-7, K12-13 | *△met12* mutants of Guy11 | This study |
| E12-8 | Ectopic transformant of Guy11 with integration of pMET12-KO | This study |
| DK7, DK13, DK18 | Double knock-out strains *△met13△met12* | This study |
| DE-1, DE-2 | Transformants of *△met12* with pMET13-KO | This study |
