# Supplementary material for: The MET13 Methylenetetrahydrofolate Reductase Gene Is Essential for Infection-Related Morphogenesis in the Rice Blast Fungus Magnaporthe oryzae
Source: PLoS One. 2013 Oct 7;8(10):e76914. doi: 10.1371/journal.pone.0076914 (PMC3792160; doi:10.1371/journal.pone.0076914)
Supplement: Table S2 — PCR primers used in this study. (DOC) [file pone.0076914.s008.doc]

**Table 2 Primers used in this study**

| **Name** | **Sequence(5′→3′)** |
| --- | --- |
| WF1 | CCGATCCAACGCAAGGTT |
| WR2 | GGATCCCCGGATATGCATTTTGAC |
| WF3 | GGATCCCAGTATAGCACTTGTCTGA |
| WR4 | AGCCGAGATGATTTGGGTT |
| F13 | CACTTCTTCTTGGCATTC |
| R13 | CCAGTCTTCA GTCGAGTG |
| HPH-BF | CTGGATCCTATTGAAGGAGCATTTTTGGGC |
| HPH-BR | CTGGATCCGCTCTTGTTCGGTCGGCATCTA |
| P13F | ACTACTGCTGCTGTTACTTG |
| P13R | TGCGTTAGTCCGAATGTCA |
| WF5 | CTGAACACTTGACCTTTC |
| WR6 | GGTACCGATGCTGATGGGCTGTGA |
| WF7 | GGTACCAATGATAAGTGTTGCGCT |
| WR8 | TCTGTATCAAGCGAGTTCT |
| Bar-KF | GGTACC AGAAGATGATATTGAAGG |
| Bar-KR | GGTACC CCTAAATCTCGGTGAC |
| P12F | CAGATGATCAAGAGGACG |
| P12R | TTGGCAGGAA CATCTCG |
| RT-1F | CCAAAGACCTCGTATCCC |
| RT-2R | GTCCTCGTCCTTGTTATCG |
| DK-R | ACCTGGTATGATGGGGAC |
| RT-3F | CTTCCCACCCAGACTACGA |
| RT-4R | CCCAAGTTGCCTCCCTAC |
| BT-F | ATTGGTGCTGCTTTCTGGCA |
| BT-R | GGAAGAGCTGGCCAAAAGGA |
| 13F | ACTTTACCCTGAACGTTG |
| 13R | AAGCTTAGACGCAGTAACAGCTG |
| GFP-13F | AAGCTTATGGTGAGCAAGGGCGAGGA |
| GFP-13R | CCGAAGCTTGTGGAGATGTGGAGTGGGCG |
| CDS13F | GGAATTCATGTTGGCCGAGACCGA |
| CDS13R | GGAATTCTCAAGACGCAGTAACAG |
| CDS12F | TCACAGCCCATCAGCATC |
| CDS12R | AGCGCAACACTTATCATT |
| 7195F | CCAAGAAGTACCTCAAGC |
| 7195R | ATCTTGAAGCCGTCCACG |
| 6712F | TACTTCGGTGACATCGTC |
| 6712R | GGCACCAACAACAGTTTC |
| 13781F | GACATTGTGACCACGACC |
| 13781R | GGGTTCTCAAGGTCGTAC |
| 383F | CCATGACTCTGCTCCTTG |
| 383R | CGTTGTCGTGCTTGTACTC |
| 4764F | AAGCTGCACCTGTTCCAT |
| 4764R | CAGAGCCTTGTCGAGATTC |
| 3583F | GATATGAGGAGGGCCAGTC |
| 3583R | CGTAATCGACGCATCTCAA |
| 923F | CGCATTGTCGGCTACTC |
| 923R | GAGGCGATAAGGATGATGG |
| 7384F | GATTGAGGAGGCCGAGAAG |
| 7384R | GAGAGCCTTGAGGACAGAG |
| 5155F | CTTCGAGGCTATGCCCAAC |
| 5155R | GGAGCAGGACATGACGAAC |
| 7074F | CGCCGATATTGTGTACGAG |
| 7074R | GCGCATGAGTAGGAAGGAG |
| 10380F | CAATCGGCACCATCACAG |
| 10380R | GCCGTACACATCCGAGAC |
| 9292F | GCCGAGACTGAAATGGTG |
| 09292R | CTGGCCATTCGTTTCTTTC |
| 10308F | TGGCATCTCGGTCTGACTC |
| 10308R | AACCGATCGTCCTCCATCC |
| 7920F | CTACATAGGCAAGTTCCTC |
| 7920R | ATGCCAGCCCGGATAGTTG |
| 6466F | CTACAAGCTCGTCATCTAC |
| 6466R | GTTGTCGAACTGGTTGGTC |
| 7195F | ACAAGCCCGAGGACCTGAT |
| 7195R | TGGGCCTGACAAAGTATCC |

Introduced restriction sites are underlined. *Bam*HI=GGATCC; *Eco*RI=GAATTC; *Hin*dIII=AAGCTT; *Kpn*I=GGTACC
